# Spatial and Temporal Resolution of Global Protein Synthesis during HSV Infection Using Bioorthogonal Precursors and Click Chemistry

**DOI:** 10.1371/journal.ppat.1005927

**Published:** 2016-10-05

**Authors:** Catherine Su Hui Teo, Remigiusz A. Serwa, Peter O’Hare

**Affiliations:** 1 Section of Virology, Faculty of Medicine, Imperial College London, St Mary’s Medical School, Norfolk Place, London, United Kingdom; 2 Department of Chemistry, Imperial College London, London, United Kingdom; University of Wisconsin-Madison, UNITED STATES

## Abstract

We used pulse-labeling with the methionine analogue homopropargylglycine (HPG) to investigate spatiotemporal aspects of protein synthesis during herpes simplex virus (HSV) infection. In vivo incorporation of HPG enables subsequent selective coupling of fluorochrome-capture reagents to newly synthesised proteins. We demonstrate that HPG labeling had no effect on cell viability, on accumulation of test early or late viral proteins, or on overall virus yields. HPG pulse-labeling followed by SDS-PAGE analysis confirmed incorporation into newly synthesised proteins, while parallel processing by in situ cycloaddition revealed new insight into spatiotemporal aspects of protein localisation during infection. A striking feature was the rapid accumulation of newly synthesised proteins not only in a general nuclear pattern but additionally in newly forming sub-compartments represented by small discrete foci. These newly synthesised protein domains (NPDs) were similar in size and morphology to PML domains but were more numerous, and whereas PML domains were progressively disrupted, NPDs were progressively induced and persisted. Immediate-early proteins ICP4 and ICP0 were excluded from NPDs, but using an ICP0 mutant defective in PML disruption, we show a clear spatial relationship between NPDs and PML domains with NPDs frequently forming immediately adjacent and co-joining persisting PML domains. Further analysis of location of the chaperone Hsc70 demonstrated that while NPDs formed early in infection without overt Hsc70 recruitment, later in infection Hsc70 showed pronounced recruitment frequently in a coat-like fashion around NPDs. Moreover, while ICP4 and ICP0 were excluded from NPDs, ICP22 showed selective recruitment. Our data indicate that NPDs represent early recruitment of host and viral de novo translated protein to distinct structural entities which are precursors to the previously described VICE domains involved in protein quality control in the nucleus, and reveal new features from which we propose spatially linked platforms of newly synthesised protein processing after nuclear import.

## Author Summary

All viruses reprogram infected cells for the synthesis, modification and targeted localisation of virus-encoded and host proteins. Advances in proteomics and mass spectrometry have provided broad insight into these processes, but these approaches have limited ability to investigate spatial aspects of infected cell protein synthesis and localisation. Here we provide the first report using novel techniques in chemical biology involving labeling newly synthesised proteins with chemically tagged amino acid precursors that enables subsequent biochemical analysis and spatial analysis by microscopy. Using these techniques, we provide new insight into protein metabolism in herpes simplex virus infected cells which is not approachable by standard methods. We report the formation of novel subnuclear domains termed NPDs (newly synthesised protein domains) with a spatial link to pre-existing nuclear PML domains and to previously described domains involved in protein quality control. This work provides new insight into metabolic processes early after HSV infection and demonstrates the considerable potential of these techniques to yield fundamental insight into virus infection and virus-host interactions in any system.

## Introduction

The manipulation of cellular metabolic processes during virus infection promotes or tempers virus production and determines the outcome of infection not only at the cellular level but also e.g., acute versus long-term persistence, latency, reactivation and transmission [1]. With regard to infected cell protein metabolism, as well as the regulated de novo synthesis of virus encoded proteins, modulation of the host proteome is necessary for both infection and host cell responses, involving modifications in protein turnover, function and location [2]. Recent advances in global proteomic approaches and mass spectrometry methods have provided broad insight into the synthesis, modification and degradation of viral and host proteins as infection progresses [3–8]. These studies reveal alterations of cellular pathways including for example, the remodeling of glycolytic and metabolic pathways [9], inflammatory and innate immune response factors [6,10] or nucleotide and RNA processing pathways [11]. However, a complete understanding of infected cell protein metabolism requires a parallel approach to spatial aspects of global protein synthesis and transport dynamics and alterations in these processes during different stages of infection. Traditional analysis of proteins at steady-state using antibodies, or fusion of genes to fluorescent proteins for dynamic spatial analysis, provide powerful tools for the investigation of individual proteins [12–14]. However, global spatial analysis requires a different approach. One method to visualise total nascent protein synthesis relies on the incorporation of puromycin, an aminonucleoside antibiotic, either using a fluorescent derivative of puromycin [15] or by the detection of polypeptide-puromycin conjugates using anti-puromycin antibodies [16]. This approach has yielded insight in the spatial analysis of cellular protein synthesis and modulation during bacterial [17] and viral infection [18]. Nevertheless there are disadvantages for spatial analysis of nascent proteins including low signal-noise ratios, qualitative differences with expected patterns [15,19,20] and importantly that puromycin is a tRNA mimetic that terminates translation, perturbing the system and eliminating the possibility of spatiotemporal analysis of fully translated proteins in e.g. pulse-chase experiments.

Advances in organic chemistry, specifically in the area of bioorthogonal ligation reactions [21] has now enabled the development of a new generation of techniques based on the in vivo incorporation of metabolic precursors that contain designed chemical endgroups. This is combined with the use of aqueous-compatible, selective covalent bond-forming reactions, commonly termed “click chemistry”. These reactions link the macromolecular products which incorporate the precursors to capture reagents via a specific paired chemical moiety [22–25]. The most routinely used chemical pairs are the azide- and alkyne moieties together with a copper-catalysed cyclization, in which the azide and alkyne form a covalent triazole ring [24,26]. The azide or alkyne groups are well suited to chemical biology since they are small, bear no overall charge, can be introduced to a wide variety of precursors and are extremely well tolerated in vivo [27–29].

Thus for protein synthesis, using the methionine analogues homopropargylglycine (HPG) or azidohomoalanine (AHA), it is possible to label newly synthesised proteins and then covalently couple fluorescently labeled capture reagents, for analysis of protein profiles by SDS-PAGE and in-gel fluorescence. At the same time, by fixing cells and then in situ coupling to similar capture agents, it is possible to simultaneously visualise the flux and intercompartmental transport of the “translatome” by microscopy [28,29]. Here we provide the first such spatial analysis of bulk newly synthesised proteins during the progression of infection with a large complex DNA virus, herpes simplex virus, providing new insight into protein trafficking. We focus on the induction of novel domains in the nucleus into which newly synthesised proteins are transported and their spatial relationship both with pre-existing PML domains and other domains, termed VICE domains, previously described to have a role in protein quality control in HSV infected cells [30–32].

## Results

### Qualitative analysis of protein synthesis by HPG pulse-labeling in HSV infected cells

**Fig 1:**
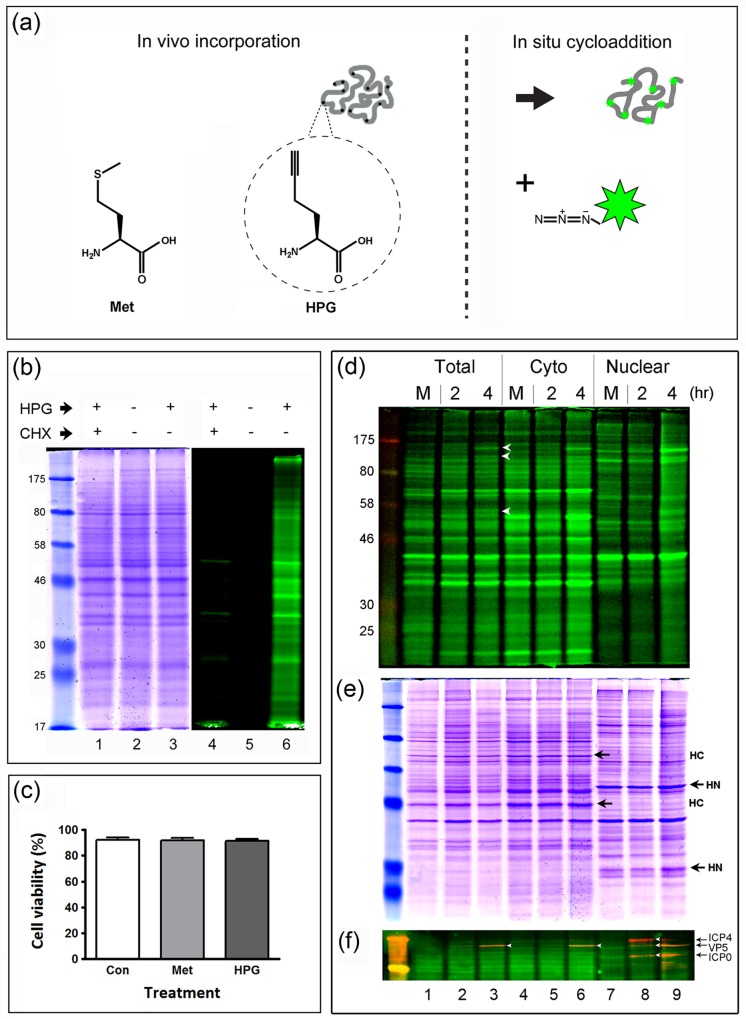
Biochemical analysis of newly synthesised proteins using HPG and click chemistry. (A) Schematic diagram illustrating comparative structures of methionine and HPG. The scheme indicates the in vivo incorporation of HPG into protein (solid black dots within a protein chain) and then the subsequent in vitro cycloaddition reaction to covalently cross link an azide fluorochrome-coupled capture reagent (coloured star) to HPG. (B) Mock-infected Vero cells were pulse-labeled using 1 mM HPG for 1 hr, lysed and subjected to click reactions using IRDye 800CW Azide Infrared Dye. Proteins were separated by SDS-PAGE and visualised by in-gel fluorescence using a LI-COR Odyssey Infrared Imaging System. Control experiments were carried out either in the absence of HPG (lane 5) or in the presence of 100 μg/ml of CHX (lane 4). Lanes 1–3 represent the total Coomassie blue staining protein profile and lanes 4–6 the in-gel fluorescence profile of the identical gel. (C) Cell viability of uninfected Vero cells (% live cells, in triplicate) was assessed by trypan blue exclusion. Control cultures were subject to either no methionine depletion and incubation in standard methionine-containing medium (Con; white bar) or methionine depletion with subsequent incubation in standard methionine-containing medium (Met; light grey bar); while HPG labelling was performed after methionine depletion with subsequent incubation in HPG -containing medium (30 min pulse). (D) In-gel fluorescence of newly synthesised proteins in total, cytosolic and nuclear fractions. Mock or HSV infected Vero cells (MOI 10) were pulse-labeled at the times indicated for 1 hr, lysed and fractionated prior to click reaction. Equal concentrations of proteins (20 μg, representing a 4-fold increased loading by cell equivalents for the nuclear fraction) were resolved by SDS-PAGE, and proteins visualised using a LI-COR Odyssey Infrared Imaging System scanned into the green channel. (E) The same gel was stained with Coomassie brilliant blue for total protein detection. Representative host cell proteins enriched within the cytoplasmic and nuclear fractions are labeled HC and HN respectively. (F) The same samples after separation by SDS-PAGE where transferred to a nitrocellulose membrane. Total steady-state levels of candidate viral proteins, ICP4, ICP0 and VP5 (red) were simultaneously detected using monoclonal antibodies, and newly synthesised proteins (green) were visualised on the blot.

Both HPG (alkyne-bearing) and AHA (azide-bearing) are structural analogues of methionine that contain inert (bioorthogonal) chemical groups that enable subsequent covalent ligation to any of a series of capture reagents. They have been shown to be non-toxic, with no effect on global rates of protein synthesis nor protein degradation. They have been evaluated by in-gel fluorescence, by in situ imaging analysis and by mass spectrometry in a variety of cell lines, in primary neuronal cells, in organotypic brain slice cultures, in developing larval zebrafish and in adult mice in vivo [33–39]. In preliminary work we compared HPG and AHA in a series of experiments for sensitivity, efficiency of incorporation and toxicity. With little difference between the two precursors, we pursued further work with HPG as the labeling precursor together with azide-linked capture reagents (Fig 1A).

We confirmed HPG incorporation into proteins using qualitative analysis by SDS-page and in-gel fluorescence. Uninfected Vero cells were labeled with 1 mM HPG for 60 min. As controls we either omitted HPG or, in the presence of HPG, included cycloheximide (CHX, 100 μg/ml) to block de novo protein synthesis. Labeled cells were lysed, subject to cycloaddition reactions to couple an azide-linked fluorochrome (IRDye.800CW) and proteins then analysed by SDS-PAGE and Coomassie staining for total protein analysis (Fig 1B, lanes 1–3) and the same gel subject to in-gel fluorescence using a LI-COR Odyssey Infrared Imaging System for de novo synthesised proteins (Fig 1B, lanes 4–6). The results demonstrate efficient labeling of uninfected cell proteins (lane 6) with essentially no background fluorescence detected in control experiments omitting HPG but samples still subject to click chemistry with the azide-fluorochrome (c.f. lanes 5 and 6). Furthermore when pulse-labeling with HPG in the presence of CHX, incorporation was virtually eliminated (c.f., lanes 4 and 6). In parallel we evaluated the effect of the identical HPG-labeling regime on cell viability (which as with standard ^35^S-Met labeling included a short methionine depletion phase, see materials and methods). We found no difference whether pulsing with HPG or methionine and neither condition had any detectable effect on cell viability (Fig 1C).

We next examined HSV infected cell protein synthesis after infection (MOI 10), pulse-labeling at early times of infection (2 hr or 4 hr), and additionally fractionating samples into cytoplasmic and nuclear samples prior to the coupling with IRDye.800CW. Furthermore, in addition to analysis by in-gel fluorescence to detect newly synthesised proteins (Fig 1D), we transferred a duplicate gel to a nitrocellulose blot (Fig 1F). This enables probing with antibodies to candidate proteins e.g., ICP4, ICP0 and VP5 (detected by secondary antibody, red channel) while bulk newly synthesised proteins were detected by the azide-coupled fluorochrome (green channel). Laser scanning of the blot allows simultaneous analysis of total active protein synthesis in one channel and of specific polypeptide abundance in a separate channel on the same blot. For the in-gel fluorescence, a number of altered species could be observed by 4 hr (Fig 1D, white arrowheads indicated on lane 3) with the fractionation procedure providing effective enrichment of individual species into cytoplasmic or nuclear fractions. Fractionation is exemplified by candidate host species (HC for cytoplasmic and HN for nuclear) in the corresponding total protein profile (panel e). We found the in-gel fluorescence of the SDS-PAGE gel itself to yield better resolved bands than fluorescence of the corresponding blots. Nevertheless, it was possible to visualise individual species on the blots, integrated with the newly synthesised population (panel f). For example, ICP4 and ICP0 can be seen by 2 hr, partitioning mainly in the nucleus (panel f, lane 5 vs 8) while VP5 was observed by 4 hr in both cytoplasm and nucleus (panel e, lane 6 and 9). Generally we found that HPG pulse-labeling and in-gel fluorescence was as good a method, in terms of sensitivity and background, as we have found in past laboratory experience with ^35^S-methionine labeling and autoradiography [40–43].

**Fig 2:**
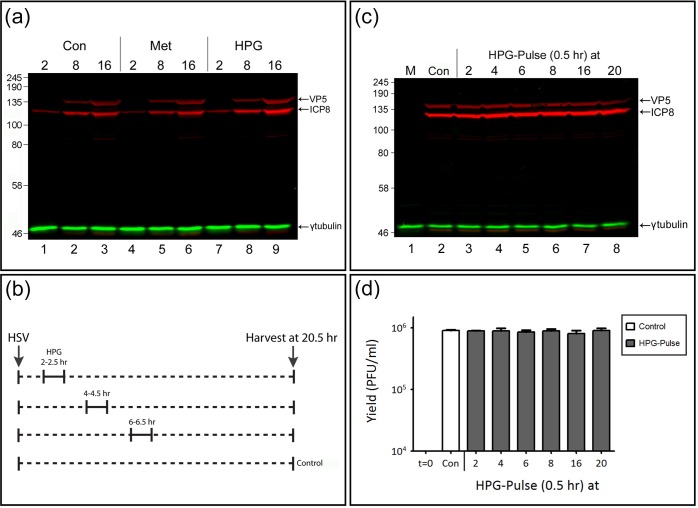
HPG incorporation has no effect on the accumulation of candidate viral proteins nor on overall virus yield. (A) Vero cells were pulse-labeled with 0.5 mM HPG for 30 min at 2, 8 or 16 hr p.i. and lysates were collected at the termination of each pulse. Control cultures were incubated as described for Fig 1 (Con and Met). Proteins were separated by SDS-PAGE and transferred to nitrocellulose membranes. Total steady-state levels of early and late viral proteins, ICP8 and VP5 (red) respectively, were simultaneously detected and γ-tubulin (green) was used as loading control. (B) Diagram illustrating the protocol for experiments in (C) and (D). Mock or infected Vero cells were pulse-labeled with 0.5 mM HPG for 30 min at 2, 4, 6, 8, 16 or 20 hr p.i. and chased in normal methionine-containing medium after removal of HPG, harvested at 20.5 hr post infection and analysed for total VP5 and ICP8 accumulation (C) and for total infectious viral yields (D).

In additional control experiments we further evaluated whether HPG pulse-labeling had any effect on the overall accumulation of virus encoded proteins, using ICP8 as a candidate delayed early protein and VP5 as a candidate late protein. Parallel cultures were either untreated (Fig 2A, lanes 1–3), subject to methionine depletion followed by pulsing with normal methionine containing medium (Fig 2A, lanes 4–6), or subject to methionine depletion followed by pulsing in medium containing HPG (Fig 2A, lanes 7–9). Cells were harvested at the various times indicated at the end of the 30 min pulse. Equal cell equivalents were then analysed for total accumulation of ICP8 and VP5. No significant differences were observed between pulsing with methionine or HPG and neither treatment had any significant effect on protein accumulation compared to the untreated control (Fig 2A). In a variation of this experiment we also evaluated whether pulse-labeling with HPG, especially at early times of infection, had any effect on viral protein accumulation later in infection or on the final yield of infectious virus. Therefore as outlined in Fig 2B, HSV infected cells were subject to methionine depletion and HPG pulse-labeling at either 2, 4, 6, 8, 16 or 20 hr post infection (p.i.). After each pulse, incubation was continued in the presence of normal methionine-containing medium up to 20.5 hr. (Nb, only the early pulse periods are illustrated on the protocol schematic, Fig 2B). Control cells were maintained in normal medium for the duration of the experiment. All cultures were harvested at 20.5 hr and analysed for ICP8 and VP5 accumulation (Fig 2C) and for the total yield of infectious virus (Fig 2D). The results demonstrate that HPG pulse-labeling had no effect on the later accumulation of test viral proteins and no effect on the overall yield of infectious virus.

Taken altogether our results are entirely consistent with previous data [33,44], which demonstrated HPG to be an effective bioorthogonal analogue of methionine, to be non-toxic, and to be incorporated into proteins with no effect on global rates of protein synthesis nor protein degradation [29,33]. We validated HPG for analysis of HSV infected cell protein synthesis showing its effective incorporated into proteins in virus-infected cells with no effect on the overall progression of infection, the accumulation of candidate viral proteins nor the total yield of infectious virus.

### Spatial analysis of new proteins synthesis

The key advantage in the use of HPG labeling of protein synthesis is the ability to undertake parallel investigation of spatial aspects of newly synthesised proteins and, in the longer term, to selectively purify newly synthesised proteins away from the total proteome based on temporal aspects of their synthesis.

**Fig 3:**
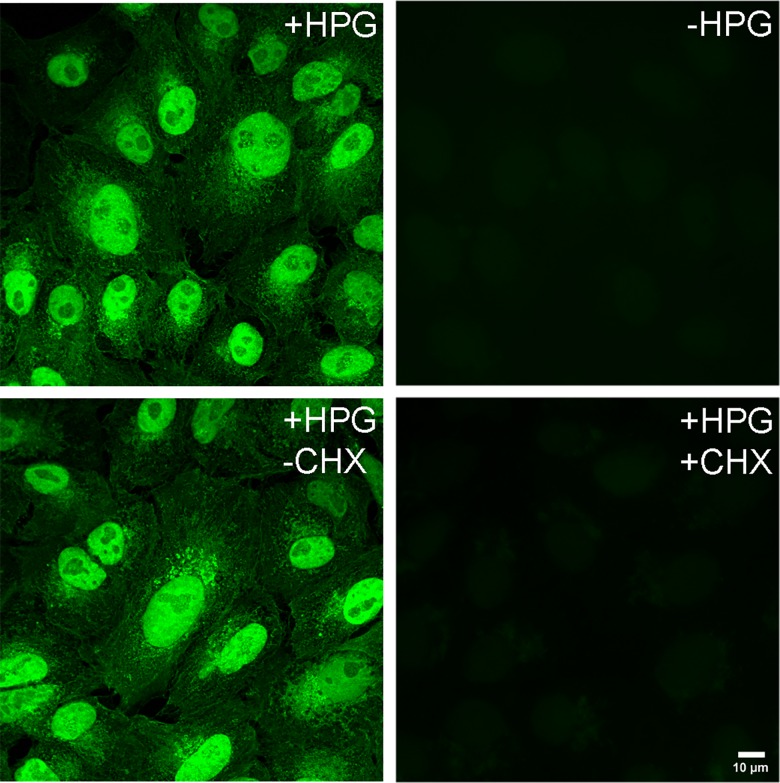
Imaging newly synthesised proteins by HPG incorporation and click chemistry. Uninfected Vero cells were pulse-labeled using 0.5 mM HPG for 30 min, fixed and subjected to click reaction using the Alexa Fluor 488-azide capture agent. Control experiments were performed either in the absence of HPG or using 100 μg/ml of CHX added prior to and during the pulse. Images were recorded as described in materials and methods.

We first pulse-labeled uninfected cells with or without HPG (30 min) and processed the monolayers for imaging analysis. As controls, we also pulse-labeled in the absence of HPG or with HPG in the presence of CHX. The results (Fig 3) demonstrate newly synthesised protein localisation in the cell with abundant accumulation in the nucleus and a disseminated lace-like pattern in the cytoplasm. There was virtually no background fluorescence observed in cells in the absence of HPG but subject to click chemistry and azide-fluorochrome detection (Fig 3,—HPG). Furthermore, in the presence of HPG together with CHX, virtually no incorporation was observed (Fig 3, +HPG,+CHX), confirming that the spatial distribution represents newly synthesised proteins, with any free HPG contributing little to the signal. These results are also consistent with previous data on the use of HPG for the spatial analysis of newly synthesised proteins in culture demonstrating both rapid import into the nucleus, relatively little free soluble cytoplasmic signal and a distinct web-like pattern in the cytoplasm likely representing association with the endoplasmic reticulum and possibly other cytoplasmic organelles [27,34].

### Nascent protein localisation to specialised sub-nuclear domains very early after HSV infection

**Fig 4:**
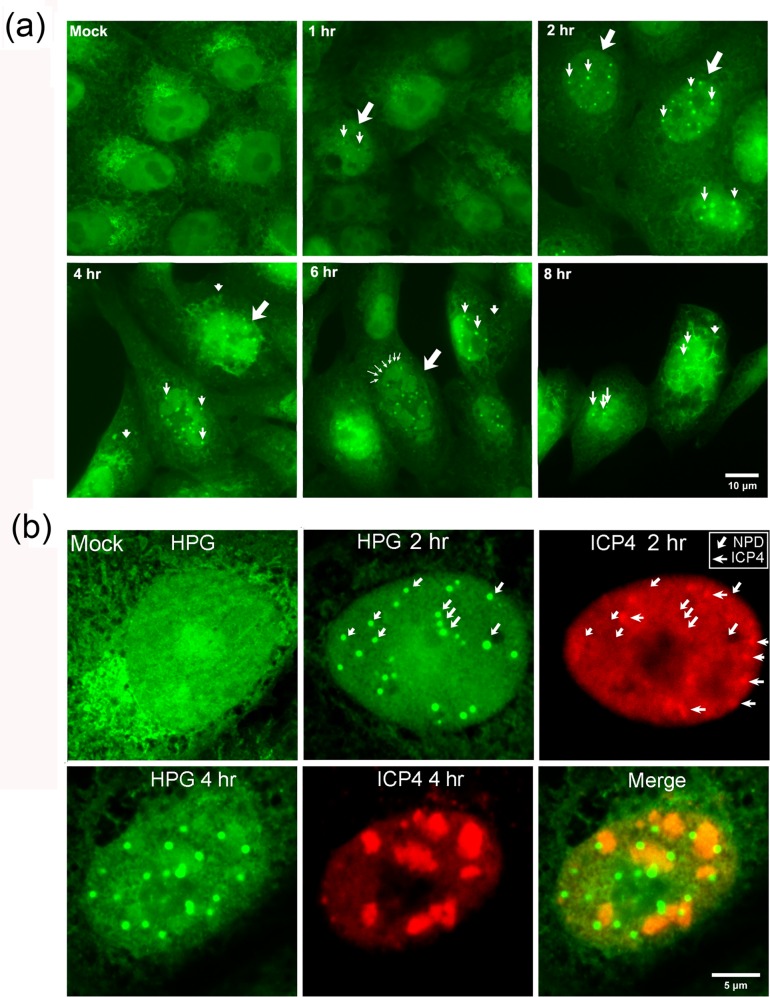
Novel nuclear NPDs revealed in HSV-1 infected cells. (A) Vero cells were mock-infected or infected (MOI 10) and pulse-labeled for 30 min at the different time points post-infection as indicated. Cells were fixed and processed as described in materials and methods. Large diagonal arrows denote nuclei containing NPDs while small vertical arrows denote the change in cytoplasmic localisation of newly synthesised proteins. (B) Higher magnification images of single cells exemplifying the spatial relationship of NPDs (green) and ICP4 (red) at 2 and 4 hr p.i. For ease of inspection separate channels are shown, with the locations of example NPDs (diagonal arrows) indicated on both channels. In the ICP4 channel, accumulation of foci of ICP4 are indicated by horizontal arrows and NPDs by diagonal arrows.

**Fig 5:**
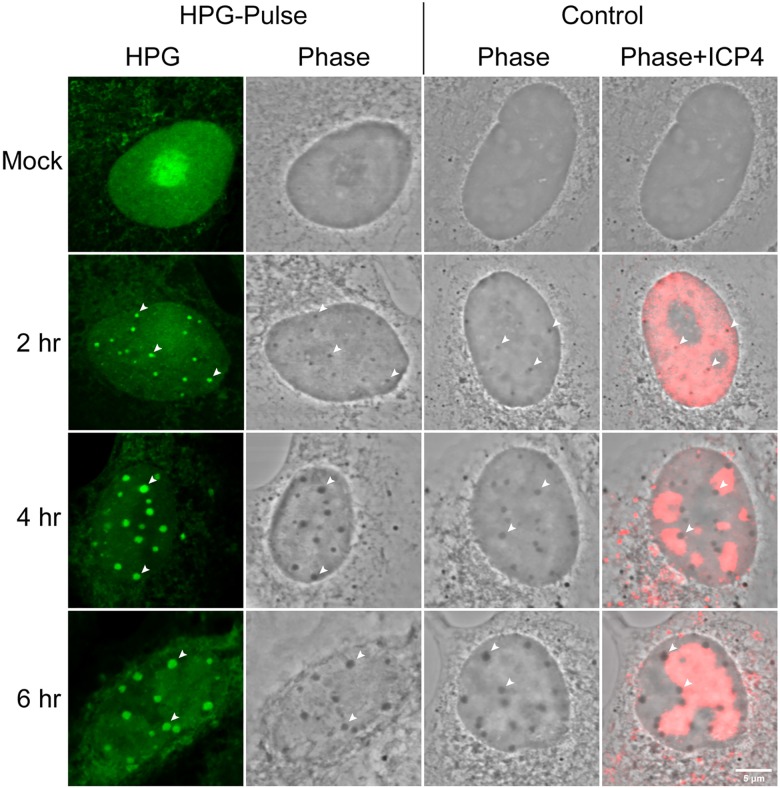
NPDs colocalise with phase-dense nuclear bodies induced by HSV. Uninfected or HSV infected Vero cells were either untreated (i.e. standard media; control) or HPG pulse-labeled for 30 min at the times indicated, fixed and analysed by fluorescence (for newly synthesised proteins, green) and ICP4 (red) and by phase microscopy. Diagonal arrowheads indicate the colocalisation of NPDs with HSV-induced phase-dense nuclear domains. Identical phase dense bodies were formed in infected cells, localising to the periphery of replication compartments whether or not the cells were pulse-labeled with HPG.

**Fig 6:**
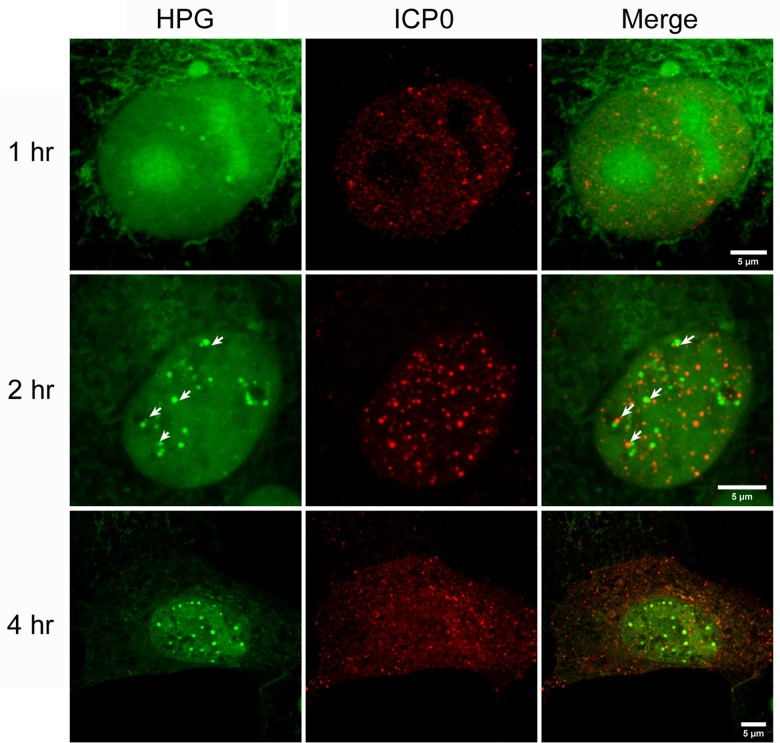
NPDs do not generally colocalise with viral IE protein ICP0. Vero cells were infected with HSV-1, pulse-labeled for 30 min with HPG at 1 2 or 4 hr p.i., fixed and analysed for ICP0 (red) and newly synthesised protein (green). Arrows point to the red ICP0 foci on the merged image and are superimposed on the HPG channel to show that the NPDs do not precisely colocalise with ICP0 foci, but rather are frequently juxtaposed.

**Fig 7:**
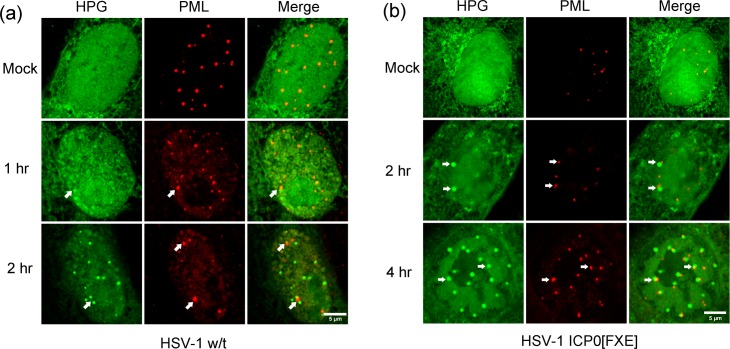
NPDs associate with PML domains in a spatially defined manner. Vero cells were mock-infected or infected with w/t HSV-1 (A) or ICP0 RING-finger mutated HSV-1 strain FXE (B), then pulse-labeled with HPG for 30 min at 1 or 2 hr p.i., fixed and processed for newly synthesised proteins (green) and total PML localisation (red). Arrows point to the PML domains and are superimposed on the HPG channel to illustrate the juxtaposition of PML to a population of NPDs for the w/t and mutant viruses as discussed in the text.

**Fig 8:**
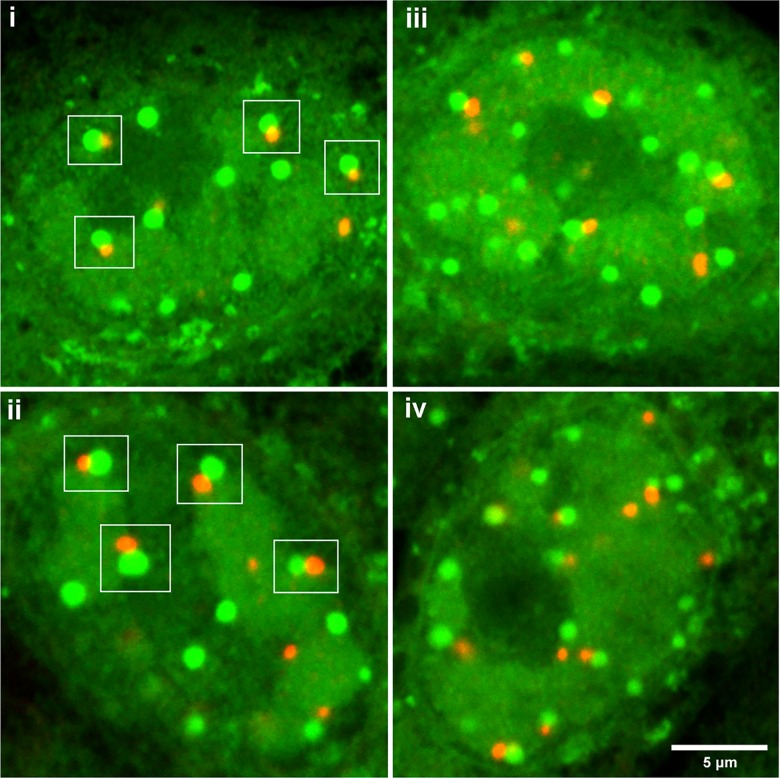
NPDs juxtapose to and overlap with PML domains in HSV-1 ICP0[FXE] infected cells. Vero cells were infected with ICP0 RING-finger mutated HSV-1 strain FXE, pulse-labeled with HPG for 30 min at 4 hr p.i. and analysed as described in Fig 7. Several typical fields are illustrated. The clear spatial relationship between PML domains and NPDs are indicated in the white squares. While many persisting PML domains were associated with NPDs, NPDs were in excess of PML domains. Quantification data of NPD/PML association as discussed in the text are presented in S1D–S1E Fig.

**Fig 9:**
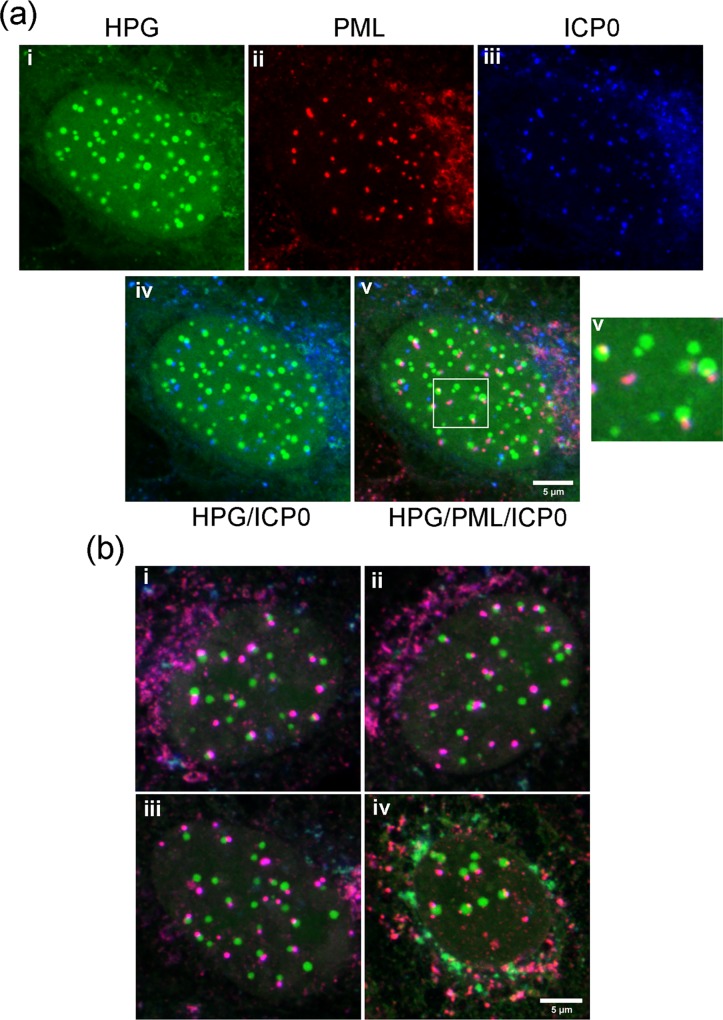
NPDs form adjacent to ICP0/PML domains. (A) Vero cells were infected and processed as described in Fig 8, in this case for simultaneous localisation of newly synthesised proteins (i, green), PML (ii, red) and ICP0 (iii, blue). The higher magnification insert emphasises the juxtaposition of PML and ICP0 in relation to NPDs. (B) Typical examples of merged channels showing precise colocalisation of mutant ICP0 with PML (purple) and the juxtaposition with NPDs (green).

**Fig 10:**
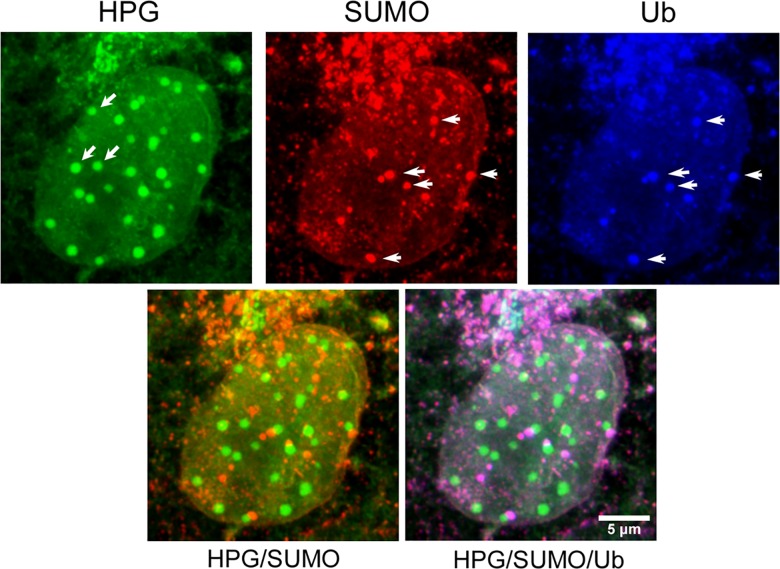
Spatial relationship between NPDs with SUMO and FK2-ubiquitin accumulation. Vero cells were infected and processed as described in Fig 8 for simultaneous detection of newly synthesised proteins (green) and total SUMO-modified (red) or ubiquitinated proteins (blue). Diagonal arrows denote example NPDs which have no obvious association with SUMO-containing foci. These latter foci do contain ubiquitinated species (horizontal arrows). For ease of inspection, the lower two fields show HPG versus SUMO alone, and HPG versus SUMO versus ubiquitin.

**Fig 11:**
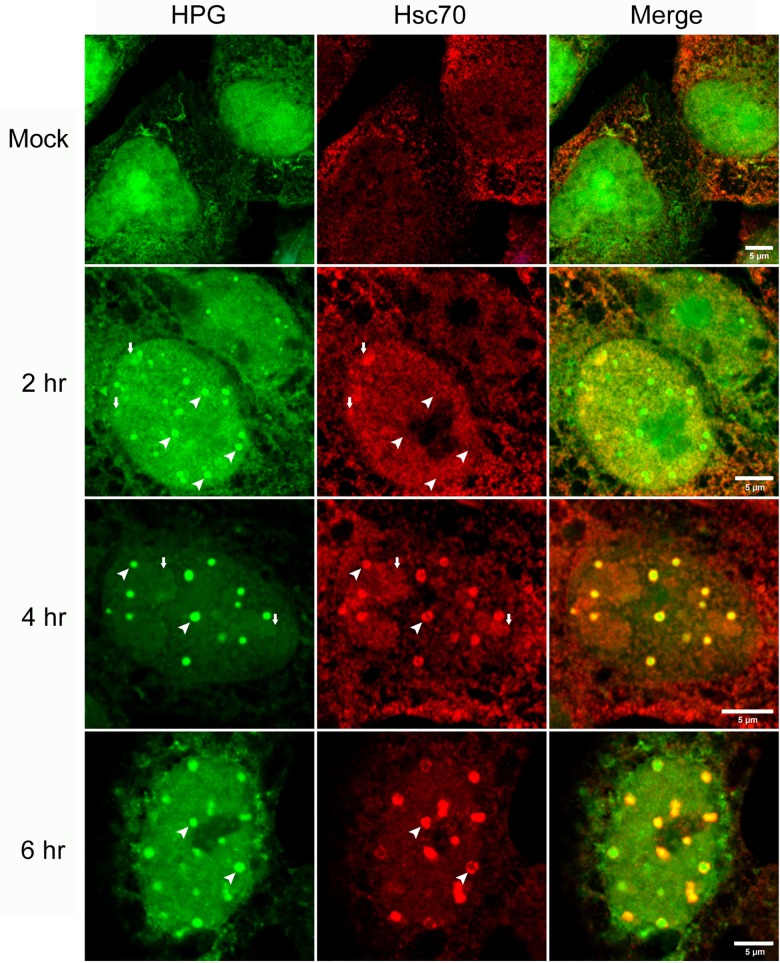
Hsc70 is recruited to NPDs at later stages of infection. Vero cells were infected with HSV-1, pulse-labeled with HPG for 30 min at the times indicated, fixed and analysed for Hsc70 (red) and newly synthesised protein (green). NPDs are indicated by diagonal white arrow heads and early in infection show no recruitment of Hsc70. Small vertical arrows indicate formation of Hsc70 aggregates, spatially separated from NPDs but also containing a population of newly synthesised protein. Diagonal arrowhead later in infection show prominent co-localisation between NPDs and Hsc70, frequently with Hsc70 coating the exterior of the NPDs.

**Fig 12:**
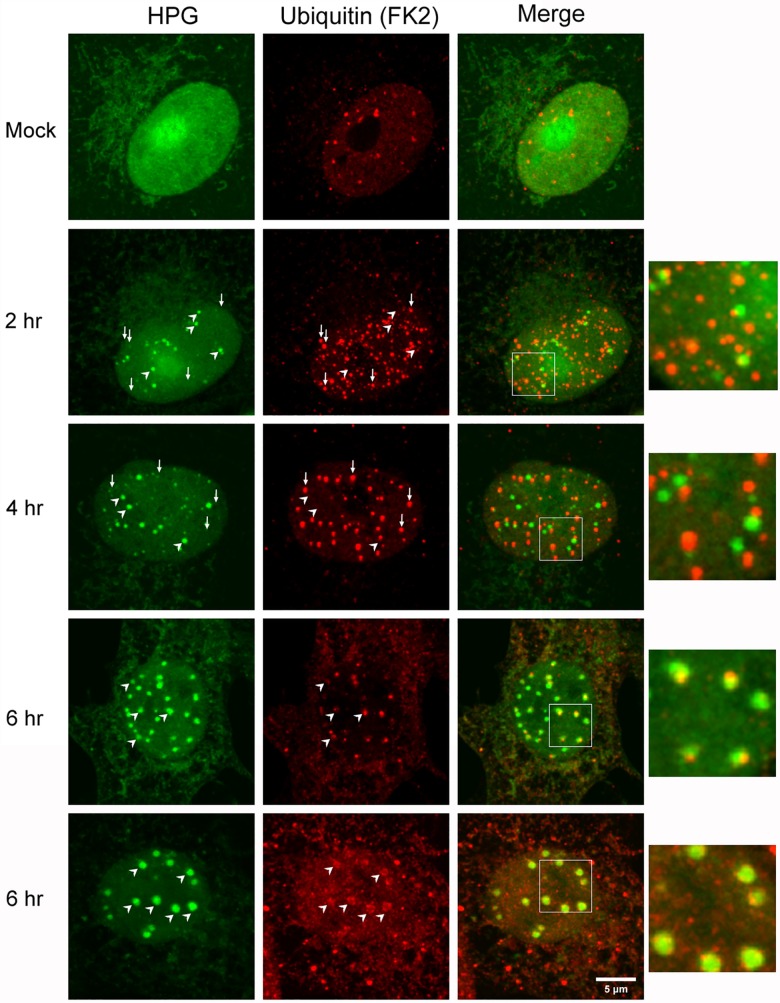
Polyubiquitinated species are recruited to NPDs at later stages of infection. Vero cells were mock-infected or infected with HSV-1, pulse-labeled with HPG for 30 min at the times indicated, fixed and analysed for polyubiquitinated species (FK2 localisation, red) and newly synthesised protein (green). In mock infected cells HPG localised in a generally diffuse pattern with some nuclear la accumulation while polyubiquitinated species were found in a speckled diffuse nuclear pattern with variable numbers of discrete foci. NPD formation in infected cells is indicated by diagonal white arrowheads and early in infection NPDs show no colocalisation with FK2+ve foci. Conversely small vertical arrows indicate FK2+ve foci which show no obvious spatial relationship with NPDs. As described in the text (see also Fig 10) a subset of NPDs localised adjacent to, co-joining FK2+ foci (see inserts of the merged fields). Diagonal arrowheads later in infection show prominent co-localisation between NPDs and FK2+ve species, either as in example cell a, as co-joining asymmetric foci or frequently, as in example cell b, with virtually complete overlap FK2+ve species coating the exterior of the NPDs

**Fig 13:**
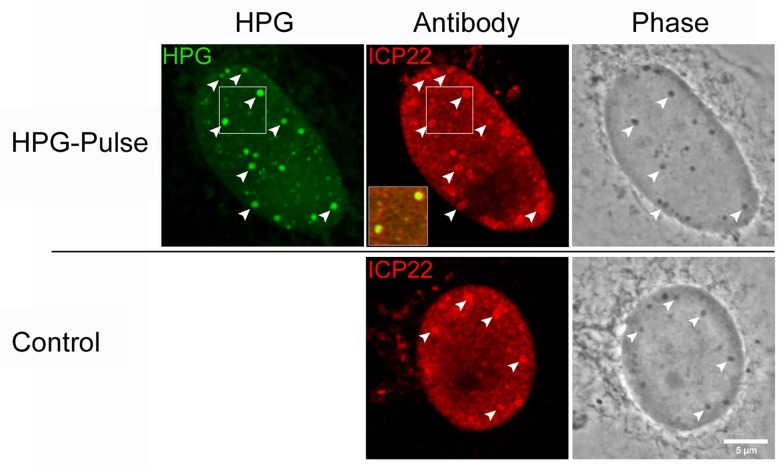
ICP22 localises to NPDs and phase-dense nuclear bodies. Infected Vero cells were either untreated (standard media; control) or HPG pulse-labeled for 30 min at 2 hr p.i. (HPG), fixed and analysed by fluorescence (for newly synthesised proteins (green) and ICP22 (red) and by phase microscopy. Diagonal arrowheads indicate the colocalisation of NPDs with ICP22 as well as phase-dense nuclear domains. The inset shows the precise co-localisation of ICP22 with NPDs. The punctate localisation of ICP22, and its recruitment into phase dense bodies was independent of HPG pulse-labeling and also observed in the control infected cultures in the absence of HPG(Control).

**Fig 14:**
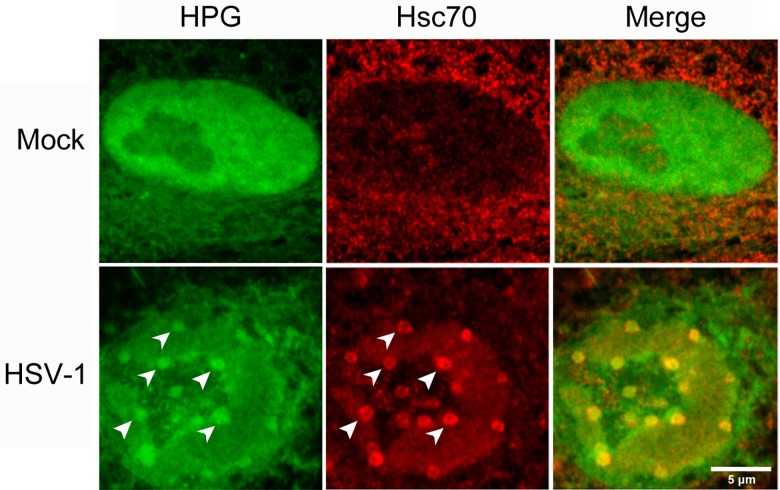
Fig Nuclear accumulation of cellular proteins synthesised prior to infection. Uninfected Vero cells were pre-labeled with HPG (30 min pulse) followed by an infection with HSV-1 at an MOI of 10 for 4 hr (HSV-1) or mock infection (Mock). Cells were then fixed and stained for Hsc70, followed by click reactions. Cells containing Hsc70 foci were identified and the subnuclear localisation of newly synthesised proteins (green) and Hsc70 (red) are shown with diagonal arrowheads indicating the colocalisation of nuclear NPDs and Hsc70 foci as discussed in the text.

We next undertook a spatiotemporal analysis of protein synthesis in HSV infected cells, pulse-labeling for 30 min at several time points up to intermediate times after infection (8 hr). While there were distinct qualitative differences in the patterns of newly synthesised protein distribution in the cytoplasm (Fig 4A, small vertical arrowheads, 4 hr and 8 hr panels), the main striking feature we observed was the formation very early in infection, within 1–2 hr, of distinct spherical domains containing HPG labeled proteins in infected cell nuclei. These domains which we have termed NPDs (newly synthesised protein domains), are indicated in a selection of nuclei at the different time points (Fig 4A, diagonal arrows) by small vertical arrows within the nuclei see also all Figs 5–14). For clarity not all domains or nuclei are labeled. NPDs were quite homogeneous in size and shape, resembling symmetrical spheres, with a consistent initial size range (mean approximately 0.5 μm).

As infection proceeded, newly synthesised nuclear proteins also localised in small irregular and aggregated clusters and then in diffuse large lobed structures distinctly resembling classical replication compartments, (Fig 4A, 4–8 hr; Fig 4B). NPDs formed early in infection and later were found specifically at the periphery of the large lobed domains. As shown below these large lobed foci, (indicated in the central cell Fig 4A, 6 hr, small arrowheads at a section of the perimeter), colocalise with ICP4 and represent DNA replication compartments [45].

Quantitative analyses of NPD formation in Vero cells are summarised in S1 Fig. Evaluating approximately 100 cells at each time point, over 50% of cells contained NPDs by 2 hr p.i. with virtually 100% positive by 4 hr p.i. (S1A Fig). In terms of NPD numbers/cell, we observed a mean of approximately 13 though some cells could contain over 25–30 (S1B Fig). Average numbers/cell showed no increasing trend as infection progressed, but the average size within a cell increased from approximately 0.5 μM diameter at 2 hr p.i. to 1.0 μM by 6 hr p.i. (S1C Fig). From quantitative analysis of the relative distribution of HPG-labeled proteins (see materials and methods), 0.5–3% of the newly synthesised protein signal in the nucleus was present in NPDs within 1–2 hr after infection, increasing progressively to as much as 10–12% later in infection.

The formation of these NPDs was observed independently of cell type and were seen with similar qualitative features after infection of lines including Vero, HaCaT, RPE, HeLa and primary MRC cells (S2 Fig).

As shown in Fig 1D and 1F, of virally encoded newly synthesised proteins within 1–2 hr only IE proteins were made to any significant extent and from quantitative scanning analysis of the lane protein profiles of newly synthesised proteins, the overall newly synthesised protein load changed little (Fig 1D, Mock vs 2 hr).

It was possible that NPDs represented new IE protein accumulation (potentially with host proteins) on infecting genomes localised in the nucleus. Therefore we examined colocalisation of NPDs with ICP4, previously shown by several laboratories to efficiently localise to nuclear viral genomes [45–47]. In contrast to our expectation, NPDs showed little localisation with ICP4. In the results (Fig 4B, 2 hr) comparing localisation of newly synthesised proteins (HPG, green channel) versus steady-state ICP4 (red channel), example NPDs (diagonal arrows) are indicated in both channels for ease of cross-reference to ICP4 localisation. The NPDs did not represent sites of ICP4 accumulation (horizontal arrows), nor were the ICP4 foci associated with NPDs. Over the course of this work, including at least ten independent experiments evaluating NPD formation versus ICP4 localisation, enumerating at least 10–20 cells in each experiment, and counting over one hundred NPDs, in no case did we observe selective ICP4 accumulation within an NPD. Rather it was very clear from the results ICP4 was selectively excluded from NPD formation.

Moreover later in infection (Fig 4B, 4 hr) as ICP4 accumulates in lobed domains which represent DNA replication compartments [45,48], we could see that NPDs did not represent sites of ICP4 accumulation and moreover were excluded from the latter, though frequently specifically abutting the ICP4+ve DNA replication domains.

### NPDs are distinct phase dense domains formed normally during infection

During our analysis we noted that NPDs could frequently be observed by phase microscopy, as small phase dense spherical domains seen exclusively in infected cells. Typical examples showing the spatiotemporal accumulation of HPG-labeled newly synthesised proteins in NPDs are shown in Fig 5 (HPG-Pulse, 2,4,6 hr). Although exceptionally a small number of phase dense domains could be observed without new protein, for the most almost all NPDs could be observed as spherical phase dense domains. We therefore examined whether such phase dense domains (which we have labeled as PDs for the purpose of cross-reference) could be observed during the progression of infection, in normal medium containing methionine in place of HPG. The results (Fig 5, Control) demonstrated that this was the case, with the timing, numbers and relative size of PDs being identical whether cells were pulsed with HPG or not. By definition we cannot analyse newly synthesised proteins in the absence of HPG but we also observed that PDs formed during infection most frequently formed outside and abutting developing replication compartments (localised here by ICP4, which is excluded from PDs). These data demonstrate not only that PD formation occurs in the natural course of infection and is unrelated to whether or not cells are pulse labeled with HPG, but also allow the conclusion that such domains represent structural sites into which a significant percentage of newly synthesised proteins in HSV infected cells is progressively recruited.

### NPDs are distinct domains spatially related to PML/ND10 domains

ICP4 was specifically excluded from NPDs and we next performed similar analyses attempting to show localisation between NPDs and ICP0 (Fig 6). Within 1–2 hr, NPDs were observed but showed no clear colocalisation with ICP0. In this case however, although it was initially difficult to attribute any specific association due to the more abundant ICP0 foci, we noted that a population of ICP0 frequently abutted NPDs (Fig 6, 2 hr; white arrows indicate ICP0 foci, in merged image and superimposed on HPG channel). As infection progressed (Fig 6, 4 hr), NPD domains were maintained while nuclear ICP0 gradually reduced as the protein became more prominent in the cytoplasm, consistent with earlier results [49–51].

The possible juxtaposition of at least a part of the NPD population with ICP0 foci led us to investigate the relationship between NPDs and steady-state PML domains (also called ND10), [52], where ICP0 accumulates at early times of infection, with resultant progressive PML domain disruption [52–55]. In mock infected cells, classical PML/ND10 domains were observed with no obvious subnuclear accumulation of newly synthesised proteins and no NPD formation (Fig 7A, Mock). Within 1 to 2 hr p.i., PML domains were progressively disrupted as anticipated [53,54,56,57]. While this made it more difficult to quantify potential colocalisation of the disrupting PML domains with the increasing formation of NPDs, nevertheless we frequently observed an association and a juxtaposition of remaining PML domains with a subset of the NPDs (Fig 7A, 1 hr, 2 hr; arrows). During high multiplicity infections with wild-type HSV, pre-existing PML domains (i.e., those present prior to infection), are disrupted and progressively disappear. Some PML protein can be mobilised or mislocalised to infecting virus genomes/protein aggregates [58–61]. However in the presence of normal ICP0, these are small foci/aggregates, discernibly distinct from original PML domains. Furthermore during high multiplicity infections with w/t HSV as performed here, any induced PML-containing aggregates are extremely difficult to discern [58,60]. Nevertheless it was possible that at least some of the NPD formation which we observed could be due to their formation adjacent to induced PML -containing aggregates. While possible, this was not the most likely explanation for our results considering the combined results of the following sections.

Thus, to investigate NPD formation further we performed a similar analysis using a HSV mutant, HSV-1 ICP0[FXE], which contains a RING-finger deletion in ICP0 and as a result does not disrupt pre-existing PML domains [46,62]. In this case, the progressive formation of the NPDs, including their localisation to the periphery of DNA replication compartments, could now be seen in clear association with PML domains, forming not in a directly colocalised manner, but rather in a juxtaposed manner directly abutting the PML domains (Fig 7B, 2–4 hr, HPG vs PML). High magnification images of several cells illustrating the typical relationship between these two structures are shown in Fig 8 i-iv and quantitative analysis of NPD formation in relation to PML domains in a population of HSV-ICP0[FXE] infected cells is supplied in panels d and e (S1 Fig). For each individual cell examined, total NPDs are indicated by green dots, total PML domains in red dots and NPD-PML associated domains (termed NPD^P^) in orange dots (S1D Fig), summarised in the bar chart (S1E Fig). On average, there were greater numbers of NPD domains in infected cells than there were pre-existing PML domains in uninfected cells with means of 15–16 and 11–12 respectively in this population. In most cells (Fig 8, i-iv), virtually all the PML domains showed a distinct spatial configuration with respect to the assembling NPDs, which formed directly adjacent and in close association, as if emanating from the PML domains. The same was not true in reverse. As indicated from the comparative numbers, other NPDs frequently formed within cells without an obligatory positional relationship with PML domains (Fig 8, i-iv; S1D and S1E Fig).

To confirm this relationship, we performed a three-way analysis simultaneously visualising newly synthesised proteins (HPG, green channel), PML domains (red channel), and ICP0 (blue channel) after infection with HSV-1 ICP0[FXE]. Various combinations of the channels are shown for ease of reference (Fig 9A, iv and v). As anticipated, and as previously shown [54,58,62], PML domains were maintained and colocalised with ICP0[FXE] protein (panels ii, iii and merged panel v). Therefore, we anticipated that NPDs would form in frequent juxtaposition to ICP0/PML and this is precisely what we observed. In particular, the HPG/ICP0 image (panels i, iii and merged panel iv) illustrates this point. A magnified section of the three colour merged panel v is also shown. Several typical examples of this three-way analysis are further shown in Fig 9B i-iv, illustrating firstly, the broad colocalisation of ICP0[FXE] and PML (red/blue overlap appearing purple) and secondly the frequent juxtaposition of NPDs (green channel) abutting PML/ICP0 domains.

### NPDs form early without accumulation of bulk sumoylated or ubiquitinated proteins

PML domains represent a major site of SUMO-1 accumulation in the cell and are also frequently found in close apposition to bulk ubiquitinated species, especially under conditions where protein degradation is blocked [63–66]. We therefore examined whether NPDs induced early in infection (4 hr post infection) represented sites of increased SUMO or ubiquitin modification. The results (Fig 10) show that while SUMO and ubiquitin colocalised in discrete foci in infected cells (Fig 10, horizontal arrows, red v blue channels), NPDs were distinct from and showed little accumulation of either SUMO or ubiquitin (Fig 10, HPG, green channel, diagonal arrows). Since PML colocalises with SUMO, and as shown above, a subset of NPDs localised adjacent to PML domains, it would be anticipated also that certain of the NPDs would localise adjacent to SUMO/ubiquitin foci and this is exactly what was observed (Fig 10, HPG v SUMO and HPG/SUMO merge).

We further investigated whether blocking protein degradation would, a) induce the formation of NPDs in uninfected cells, or b) affect their formation in HSV infected cells. We therefore examined the effect of addition of MG132 (added prior to and during the HPG pulse) on protein localisation in uninfected and HSV infected cells in relation to PML domains. The results show MG132 treatment produced no qualitative effect on protein localisation in uninfected cell nuclei (S3A Fig, green channel), while at the same time, consistent with previous reports [64], increased numbers of PML domains were observed (S3A Fig, red channel).

In HSV infected cells (S3B Fig), as previously shown [54,67,68], MG132 blocked the disruption of PML domains resulting in more numerous domains than those seen in untreated uninfected cells (S3B Fig, c.f., PML -/+ MG132). MG132 treatment also resulted in increased numbers of NPDs in infected cells. The spatial and numeric relationships between NPDs and PML domains in infected and MG132 treated cells were complex as described below.

Thus, PML localisation could for the most part be categorised into one of two types. Type 1 domains (S3B Fig, PML channel, vertical arrows) were generally more homogeneous and spherical, and resembled the domains normally seen in uninfected cells. Type 2 domains (diagonal arrows) were generally larger, irregular and more aggregated in appearance. This grouping could be made on the basis of morphology with anti-PML antibody alone but was reinforced by a clear selective association of the type 2 PML domains with a subset NPDs. Thus while type 1 PML domains showed little accumulation of newly synthesised proteins and no obvious association with NPDs, type 2 PML domains showed a clear association, often a completely overlapping colocalisation with NPDs (S3B Fig, +MG132; diagonal arrows, HPG vs PML). The expanded insert (S3B Fig, +MG132, centre field), illustrates in each channel, adjacent type 1 (lower domain) and type 2 PML domains (upper domain), with only the type 2 domain exhibiting distinct colocalisation with a NPD. Possible explanations are proposed in the discussion. Thus Type 1 PML domains could represent original “older” domains, present before treatment with MG132, which as shown above (Fig 7), do not accumulation newly synthesised proteins. Type 2 PML domains would then represent those domains induced specifically after proteasome inhibition, with these domains accumulating newly synthesised proteins made during the pulse timeframe after drug treatment. Although the spatial and temporal relationship is complex, taken together with results of the previous section, several points are clear; 1) that proteasome inhibition per se was insufficient to induce NPDs in uninfected cells, 2) that HSV infection was required and 3) that NPDs very frequently accumulated in a precise spatial relationship co-joining pre-existing PML domains.

### Formation of NPDs requires viral gene expression but is independent from DNA replication and late gene expression

We next used inhibitors to examine what broad phases of events were required in infected cells to induce NPDs. Clearly by definition, inhibition of protein synthesis with CHX completely blocked NPD formation (Fig 2). Consistent with their formation very early after infection, inhibition of DNA synthesis by acycloguanosine (S4 Fig, Con vs ACG) had little effect on the formation of NPDs. Blocking de novo infected-cell transcription with actinomycin D (Act. D) prevented the formation of NPDs indicating that virus infection per se did not result in the accumulation of any newly synthesised cellular proteins (translated in the presence of Act. D) into NPDs. Interestingly however, inhibition of transcription did result in the accumulation of newly synthesised cellular proteins in a very distinct manner, colocalising around cellular nucleoli (S4 Fig). It is well known that Act. D specifically induces the formation of “nucleolar caps” [69], representing the relocalisation of nucleolar proteins within a disrupted nucleolus together with the recruitment of novel nucleoplasmic proteins to the caps [70,71]. Our results are consistent with these previous data, reflecting the recruitment of newly synthesised proteins (nucleoplasmic and nucleolar) to Act. D-induced caps. These structures however are unrelated to NPD formation. Our analysis of protein localisation in the presence of Act.D indicate perhaps not unexpectedly, that virus gene transcription is required for NPD formation.

We also tested whether NPDs could be observed in uninfected cells undergoing various stresses or treatments, including proteasome inhibition, heat shock and interferon treatment (S5A–S5C Fig). However while these various conditions produced the expected phenotype, e.g. an increase in SUMO/ubiquitin foci with MG132 treatment [64], localisation of Hsp70 in the nucleolus during heat shock [72–74] and an increase in PML domains after interferon treatment [75,76], under none of these conditions did we observe formation of the typical NPDs in uninfected cells that we observe in HSV-infected cells (S5 Fig).

### Relationship of NPDs to VICE domains

A number of studies including from our own laboratory, have previously reported on the localisation of individual viral encoded proteins or GFP-fusion proteins to small spherical domains within the nucleus of HSV infected cells [77–80]. In particular we noted the potential similarity between NPDs and previously described virus induced chaperone enriched (VICE) domains [31,80,81]. These domains were reported to form progressively during HSV infection at the periphery of late replication compartments and to recruit certain components of the protein quality control machinery, with the defining feature being the recruitment of Hsc70 and associated co-chaperones [30,31,82]. However there were certain differences in the characteristics of NPD formation reported here and VICE domain formation characterised in previous reports. For example, we observed efficient NPD formation during infection with an ICP0-defective mutant while VICE domain formation (i.e., Hsc70 recruitment) was significantly suppressed [81]. Furthermore while NPDs did not initially colocalise with abundant Sumo or ubiquitinated species, VICE domains recruit significant levels of ubiquitinated species. Nevertheless given the similarities in relative spatial localisation we investigated the relationship between NPDs and VICE domains using the diagnostic marker Hsc70. The results (Fig 11) indicated that there was indeed a clear spatial relationship between NPDs and VICE domains but also a distinct temporal separation in their formation.

Thus, at very early times after infection NPD formation could be readily observed without any recruitment of Hsc70 (Fig 11, 2 hr). We noted occasional localisation of Hsc70 with more irregular, aggregated HPG-containing foci (Fig 11, 2 hr, small vertical arrows). These latter aggregates however were distinct from the defined regular spherical NPDs which showed no enrichment nor any recruitment of Hsc70 (Fig 11, 2 hr, large arrowheads). By contrast, at later times of infection, Hsc70 could now readily be seen to be recruited to defined NPDs, frequently appearing as a coat, surrounding the outside of the NPDs. Representative images (Fig 11, 4, 6 hr) show many NPDs by these stages contained significant levels of colocalised Hsc70, with quantitative analysis demonstrating progressively increasing Hsc70 association with time (S1 Fig, panel f).

VICE domains, in addition to recruitment of Hsc70, have been reported [81] to recruit ubiquitinated proteins as detected by the monoclonal antibody FK2 [83]. However the results of Fig 10 indicated that, at least during early times of formation, NPDs showed no selective recruitment of polyubiquitinated species, although a subset of NPDs were found frequently directly adjacent to ICP0/PML/FK2 positive foci. Considering the temporal distinction between NPD formation and Hsc70 recruitment, we evaluated the temporal recruitment of polyubiquitinated species with results indicating broad similarity to Hsc70 (Fig 12). Thus, by 2 hr postinfection, numerous NPDs were observed without any co-localisation with polyubiquitinated species (which were found in variable numbers of discrete independent foci, as well as a diffuse speckled background pattern very similar to uninfected cells (Fig 12, 2 hr). In the figure, vertical arrows indicate pronounced FK2+ve foci which show no obvious relationship with NPD formation, while diagonal arrowheads show NPD formation without significant recruitment of polyubiquitinated species. The insert shows that a subset of NPDs could be found with co-joining FK2+ve foci consistent with earlier results (Fig 10). As infection progressed the increasing presence of polyubiquitinated species with NPD domains could be observed, either as more obviously co-joining foci (Fig 12, 6 hr, a) or with more complete co-localisation with FK2 localising in a rim like pattern around the outside of the later NPDs (Fig 12, 6 hr, b), a pattern very similar to that seen for Hsc70. Generally it appeared that Hsc70 recruitment to NPDs could be seen more readily earlier than the FK2+ve staining, though this may reflect differences in antibody detection. What was clear that NPDs formed very early without any significant recruitment of either species.

To pursue further the association between NPDs and VICE domains, we examined the relative localisation between NPDs and ICP22. As shown above (Fig 4) ICP4 did not colocalise with NPDs while a population of ICP0 localised within ND10 domains frequently adjacent to NPDs (Figs 7 and 8). ICP22 on the other hand has been reported to be precisely localised to VICE domains, colocalising with recruited Hsc70 [32]. The results (Fig 13) demonstrated the efficient recruitment of ICP22 to NPDs even at early times after infection prior to Hsc70 recruitment (2 hr), and as with Hsc70 at later times, frequently coating the perimeter of the NPDs. Both the NPDs and colocalising ICP22 could be observed as phase dense bodies (Fig 13, arrowheads). We note that the localisation pattern of ICP22 and its recruitment to phase dense bodies was observed whether or not cells were labeled with HPG (Fig 13, control).

Altogether these results reconcile our earlier data and reinforce the conclusion that NPDs form early in infection in the absence of pronounced recruitment of Hsc70 or ubiquitinated species. Based on these results, differences in characteristics of the formation of NPDs and VICE domains can now be explained in the model, as outlined in the discussion, whereby NPDs recruit de novo synthesised proteins into specialised physical subdomains which are precursors to VICE domains and later through qualitative or quantitative alterations progressively recruit Hsc70 and related factors.

### Nuclear accumulation of cellular proteins synthesised prior to infection

To examine whether NPD/VICE domain formation included the recruitment of host cell proteins made prior to infection we performed a pulse-chase experiment whereby uninfected cells were pre-labeled with HPG prior to infection (30 min pulse), the label washed out and cells then infected in the presence of normal methionine. In this type of pulse-chase regime, from the time of infection onwards newly synthesised virus or host proteins would not be labeled, and localisation would track only those proteins synthesised in uninfected cells, prior to infection. In this case, we examined NPD/VICE domain using progression to Hsc70 recruitment as a diagnostic marker. The results indicated that in virtually all Hsc70-containing domains, recruitment of host cell proteins made during the labeling period prior to infection was observed. A typical field is shown in Fig 14, with pre-labeled protein recruited into foci surrounded by Hsc70 in a coat-like manner. This contrasted with the localisation in uninfected control samples that were pulse-labeled and then chased for a similar period (Fig 14, Mock). We noted also in infected cells that had not progressed to form Hsc70-containing domains, that the distribution of the previously synthesised cellular proteins could also be observed in more irregular foci and speckles, frequently at the nuclear periphery. These results indicate that the detailed kinetics of recruitment of that cohort of cellular proteins made prior to infection could differ from those cellular proteins made early after infection. Nevertheless it was clear that a significant population of cellular proteins translated just prior to infection were subsequently recruited into NPD/VICE domains as infection progressed.

## Discussion

We employ emerging techniques in chemical biology to provide the first spatiotemporal analysis of newly synthesised proteins during infection by a human virus, herpes simplex virus. We validated the use of the methionine analogue, HPG, for analysis of protein synthesis by SDS-PAGE and in-gel fluorescence, confirming HPG incorporation into bulk newly synthesised protein species. Our results are consistent with previous reports on HPG incorporation and click chemistry for the analysis of protein synthesis in tissue culture cells, which demonstrated also that HPG was incorporated in an unbiased fashion, non-toxic and did not alter global protein synthesis or turnover kinetics [33,44]. We found the resolution of the production and timing of virus induced proteins by HPG-labeling, SDS-PAGE and in-gel fluorescence to compare favourably with that we have routinely obtained in our laboratory by ^35^S-methionine pulse-labeling and autoradiography [40–43,84,85].

### Spatial analysis of newly synthesised proteins

Our results on spatial analysis in uninfected cells show the rapid accumulation of newly synthesised proteins into the nucleus as well as their distribution into cytoplasmic membranous compartments which, from co-localisation with steady state markers (S6 Fig) represent newly synthesised proteins partitioning to ER, Golgi and other organelles. These results are consistent with and expand upon previous analysis of newly synthesised proteins in uninfected mammalian cells [20,33,44,86,87]. They also indicate that by varying the duration of HPG pulse-labeling combined with different chase times in normal medium, it should be possible in future work to investigate cytoplasmic protein flux through the protein export and compartmentalisation apparatus and the perturbation of those processes as infection precedes.

### Specialised domains of newly synthesised proteins in the nucleus

**Fig 15:**
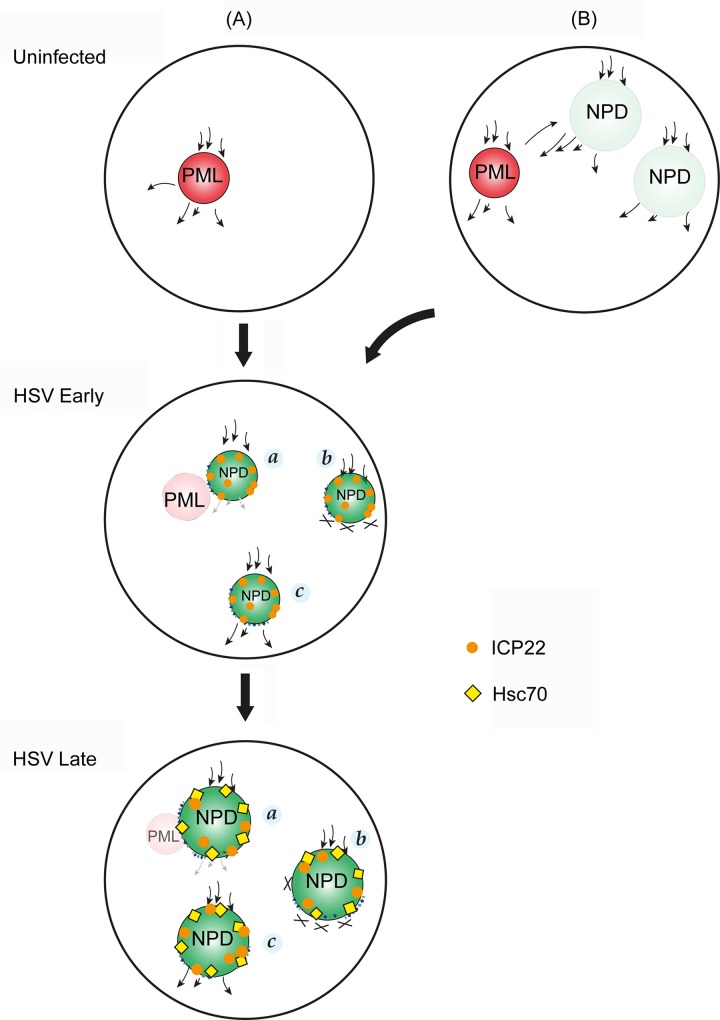
Schematic model of the spatial relationship between NPDs, PML domains and VICE domains and potential processing pathways of newly synthesised proteins. The dark red shading indicates PML domains in uninfected cells and their functioning with dynamic recruitment and dissociation of target proteins (arrows in and out). The transparent lighter red shading in HSV infected cells indicates their progressive structural and functional disruption. NPDs are formed de novo only after infection (A) or in model (B), the possibility of pre-existing functional NPDs is indicated. Like PML domains, these could represent sites of dynamic protein recruitment and onward transport. As such they would not accumulate bulk newly synthesised proteins, and are thus indicated with transparent, light green shading. Their functional disruption, or overload, in infected cells is indicated by the dark green shading, firstly as smaller domains recruiting selectively ICP22, and only later in infection recruiting additional proteins including components of the host cell protein quality control machinery and in particular Hsc70, the diagnostic marker of VICE domain formation. As discussed in the text, small arrows (NPD,a) indicate impaired forward transport from NPDs and crosses (NPD,b) indicating a more complete block of transport of client proteins after continual recruitment. NPD,c, indicates a more pronounced forward transport after recruitment of certain classes of client proteins. ICP22 (magenta sphere), but not Hsc70, are recruited to NPDs during early stage of infection. As infection progresses, bulk Hsc70 (yellow diamond), and polyubiquitinated species together with ICP22, accumulates at NPDs of larger size.

Here we focus on nuclear protein localisation and the assembly of novel domains in infected cell nuclei which we have termed newly synthesised protein domains (NPDs). NPD formation was observed within 1–2 hr of infection when only the viral IE proteins were expressed to any significant extent and when there was little global change in the overall rate of protein synthesis or load compared to uninfected cells (see Fig 1D and 1F). We initially considered that NPDs may represent sites of protein accumulation, including ICP4, on infecting virus genomes but this was clearly not the case. Rather we show a distinct spatial association between NPDs, PML domains and virus-induced VICE domains and propose a model for protein processing in the infected cell nucleus which links these observations together (Fig 15).

This model illustrates the outcome of wild-type HSV infection and attempts to unify observations: i) on the induction of NPDs and parallel disruption of PML domains, ii) the frequent but not exclusive association between PML domains and NPDs under conditions where PML domains persist (e.g., with an ICP0 mutant) and iii) on the relationship between NPD formation and VICE domain formation.

NPDs are proposed to be absent from uninfected cells and are only induced following infection (Fig 15A). However it is conceivable that NPDs could be present in uninfected cells (Fig 15B) but for example, function normally in terms of protein flux, and therefore not be detected by protein accumulation and without prior knowledge of stable structural components would therefore not be detected. PML domains (Fig 15, red spheres) comprise proteins with a more stable structural role, together with proteins in dynamic flux of recruitment and dissociation [75,88,89] (Fig 15, indicated by directional arrows)Vero. However these domains are detected solely from the point of view of having antibodies (or targeted chimeric fluorescent proteins), to visualise the steady-state localisation of key constituents including e.g., PML or SP100.

In the pathway for de novo formation following HSV infection (Fig 15A), arrows indicate the recruitment of newly synthesised proteins into the NPDs, likely representing a significant population of newly synthesised nuclear proteins. There is clear selectivity in recruitment to NPDs. PML domains which are generally of similar size and morphology to NPDs, do not detectably accumulate abundant newly synthesised proteins. Analysis of the distribution of HPG containing proteins in NPDs versus the total nuclear population during the pulse period indicates that 1–3% of the labeled nuclear protein could be detected in NPDs within 1–2 hr p.i. Parallel analysis of the distribution of these same proteins by in-gel fluorescence, indicates that the overall newly synthesised protein load in the nucleus changed only marginally, representing at this stage mainly host cell proteins imported into the nucleus and comparatively low overall abundance viral proteins, mainly of the IE class (Fig 1D, c.f. lanes 1 and 2 or 7 and 8). Neither ICP4 nor ICP0 were recruited into NPDs (although as shown above ICP0 was frequently in juxtaposition due to its association with PML domains), while early in infection ICP22 was selectively recruited. Considering the fraction of the total de novo synthesised nuclear protein recruited, the implication is that the majority of newly synthesised proteins within NPDs initially represents host cell proteins, together with ICP22 and that they progressively recruit additional viral and host proteins (see below). This conclusion is consistent with our pulse-chase analysis with pre-labeled uninfected cells showing recruitment of what must be exclusively newly translated host cell proteins into later NPD/VICE domains.

The distinct spatial relationship between PML domains (under conditions of persistence) and NPDs warrants speculation on a functional relationship between the two. One possibility is that the recruitment rate of proteins to NPDs is significantly higher than their dissociation rate, resulting in the appearance of these structures (Fig 15, HSV, NPD *(a*,*b)*). PML disruption could then be an upstream event, causing e.g. a bottleneck in a spatially and functionally linked processing pathway involving the two types of domains. However, PML disruption is not required for NPD formation (since the latter are still formed after infection with the ICP0 mutant where PML domains persist), and while PML domains were almost always coupled to NPD domains, the same was not true in reverse with NPDs frequently forming without a clear association with persisting PML domains. Indeed the converse, that NPD formation was causally linked and upstream of PML disruption is also plausible, e.g. representing a block in the onward transport of proteins from NPDs to PML domains. In this case again however since NPDs and PML can be observed together after infection with the ICP0 mutant, this proposal would require that NPD formation was linked to but not sufficient for PML disruption.

As indicated above one related possibility, still maintaining a link between the two domains, is that NPDs pre-exist in uninfected cells and like PML domains comprise both structural components and sites of dynamic protein trafficking (Fig 15, Uninfected B). Here the proper functioning and flux of proteins (signified by light shading) would mean that they would not accumulate bulk nuclear protein and without any specific antibody with which they are marked, NPDs would not be visualised. There could be trafficking exchange between NPDs and PML domains, whether or not they were in close physical association. If NPDs did pre-exist then our model would propose that rather than de novo assembly, HSV infection would perturb the normal flux through these pre-existing domains, revealing their presence adjacent to PML domains with which they may be already linked.

### NPDs in relation to VICE domains

Previous work has demonstrated that HSV infection induces the formation of discrete domains adjacent to and at the periphery of virus replication compartments [81]. These sites were shown to recruit host proteins of the quality control machinery including Hsc70 and contain ubiquitinated proteins and components of the 20S proteasome [31,81] while in other work, ICP22 and potentially ICP27 were also shown to be required for Hsc70 relocalisation into such foci [32,90]. It has been proposed that these domains, termed VICE domains, (virus-induced chaperone-enriched domains) represent sites of nuclear protein quality control that may aid in protein folding or remove aberrantly folded proteins to promote infection [30,31]. Indeed drug-mediated inhibition of chaperone function or the presence of a dominant negative Hsc70 mutant have both been reported to reduce HSV replication [80,90].

Considering our cumulative data on NPD formation and protein localisation together with previous characterisation of VICE domain formation we can propose a model that reconciles any differences between the two, whereby early in infection de novo synthesised host cell proteins together with in particular, ICP22 and possibly ICP27 [90] are recruited to NPDs which form the precursors to VICE domains. At this stage neither later virus encoded proteins, such as UL6 [81] or VP13/14 [91,92] nor host proteins e.g., Hsc70 are recruited to NPDs. In this model NPDs are still formed efficiently in the absence of functional ICP0, but because ICP0 is required for normal robust later protein synthesis, in the absence of ICP0 the early formed NPDs would not progress and would not recruit additional proteins including Hsc70. Since Hsc70 recruitment (rather than formation of domains containing new synthesised proteins) is the definition of VICE domain formation, this model therefore adequately reconciles the apparent difference in requirement for NPD and VICE formation. However we find no support for the suggestion that NPDs/VICE domains are formed from components of PML domains [93] and as stated we do not find that NPDs are formed mainly after PML domain disruption, although with Hsc70 recruitment as the hallmark, this does usually occur temporarily later, after PML disruption.

Our results provide a linking explanation for the formation of VICE domains and their progressive recruitment of protein quality control machinery. We propose earlier in infection, defined and structurally organised domains (as for PML domains) are formed into which significant levels of de novo translated host,-and later viral cellular proteins are recruited. Whether or not these domains pre-exist in uninfected cells will require structural characterisation and protein identification, at least of proteins which would be more stably present perhaps forming some sort of scaffold but our current favoured model is that NPDs form de novo during infection. At least a subset of these NPDs could be present as a spatially linked protein processing platform with PML domains, with proteins dynamically recruited to and trafficking onward from NPDs. It is also possible that NPDs represent a processing or folding bottleneck and their formation (Fig 15, HSV NPDs labeled a and b), as previously suggested [30,31,80], acts as a cyto-protective mechanism to prolong the viability and functioning of infected cell viral protein production and assembly or as an intermediary quality control site from which properly folded and assembled subunits would be released after appropriate monitoring mechanisms, to form functional assemblies e.g. multimeric or multi-subunit complexes (Fig 15, NPD, domain labeled c). From the combined analysis of our data we believe that a significant fraction of newly translated proteins that are imported into the nucleus, traffic into or through NPDs. Is likely that for individual proteins or assemblies there are differences in the kinetics of trafficking onward from NPDs. Individual species of proteins that have slower kinetics (or indeed are retained for prolonged periods) would then more readily register in NPDs by steady-state analysis using antibodies and conventional immunolocalisation.

Ultimately a complete characterisation of NPDs will require biochemical, approaches which are tractable since newly synthesised proteins incorporating HPG can be selectively purified away from the total proteome by chemical coupling to other capture reagents, allowing the integration of spatiotemporal data with analysis on biochemical fractionation, enrichment and mass spectrometry of newly synthesised proteins [33]. While such work is beyond the scope of this current analysis which focuses on spatial aspects of bulk protein dynamics early in infection, our results provide novel insight into fundamental processes in protein metabolism and trafficking early after HSV infection and possible unifying explanations for other observations in the field.

## Materials and Methods

### Cell culture

African Green Monkey kidney fibroblast (Vero) cells, human epithelial keratinocytes (HaCaT), human fetal lung fibroblast (MRC-5) cells and HeLa cells were grown in Dulbecco’s Modified Eagle’s Medium (DMEM; Gibco) supplemented with 10% Fetal Bovine Serum (FBS; Gibco), and penicillin/streptomycin (Gibco). RPE-1, a human telomerase immortalised retinal pigment epithelial cell line, was grown in DMEM/F12 (Sigma) supplemented with 200 mM glutamine, 10% FBS and penicillin/streptomycin.

### Viruses, infections and treatments

The viruses used in this study were HSV-1[17] and ICP0 ring finger-mutated HSV-1 strain FXE (kindly supplied by Professor R. Everett). Infections were routinely performed at a multiplicity of infection (MOI) of 10. Infectious viral titers were determined by plaque assays on Vero cells. For the examination of the effects of HPG pulse-labelling on virus yields (Fig 2), supernatants were collected either immediately after inoculum removal (t = 0) and an acid wash (40 mM citric acid, 10 mM KCl, 135 mM NaCl, pH 3) to represent virus input or at 20.5 hr p.i., to represent the final yields. In studies of NPD formation in the presence of various inhibitors, ACG (Thermo Scientific) was used at a final concentration of 10 μM for the inhibition of viral DNA synthesis, and Act. D (Sigma-Aldrich) was used at 5 μg/ml for the inhibition of transcription. MG132 (Calbiochem) at 10 μM was added to uninfected or infected cells for 4.5 hr before analysis. For heat shock of uninfected cells, monolayers were transferred from 37°C to a 42°C incubator for 1 hr prior to pulse-labeling (heat treatment continued during labeling) and fixed immediately for analysis. Interferon-αA/D (Sigma-Aldrich) at 5000 U/ml was added to uninfected cells for 6 hr before pulse-labeling and processing.

### Antibodies for immunofluorescence studies

The following antibodies were used: mouse anti-ICP4 MAb (Virusys) (1:500); mouse anti-ICP0 MAb (Virusys) (1:300); rabbit anti-ICP0 (r190) serum (kindly supplied by Prof. R. Everett) (1:500); rabbit anti-ICP22 serum (kindly supplied by Prof. S. Rice) (1:400); mouse anti-PML MAb 5E10 (a kind gift from Dr. R. van Driel) (1:10); rabbit anti-SUMO polyclonal antibody (PAb) (Alexis Biochemicals) (1:100); mouse anti-ubiquitin FK2 MAb (Affiniti Research) (1:600); rat anti-Hsc70 MAb (Abcam) (1:75) and mouse anti-HSP70 MAb (Sigma) (1:200). Goat anti-rabbit Dylight 594 immunoglobulin G (IgG) (1:500) was obtained from Pierce Thermo Scientific. Alexa Fluor 635 Goat anti-mouse IgG and Alexa Fluor 546 Goat anti-mouse IgG were used at a 1:500 dilution and were purchased from Molecular Probes. FluoProbes 547H Donkey anti-rat IgG (1:400) was obtained from Cheshire Sciences.

### Immunofluorescence studies

For immunofluorescence analysis, cells on glass coverslips were fixed at times indicated in 4% paraformaldehyde for 10 min, permeabilised with 0.5% Triton X-100 (Sigma) for 5 min, and blocked with phosphate-buffered saline (PBS) containing 10% goat serum for 30 min at room temperature (RT). Cells were immunolabeled for 1 hr at RT with primary antibodies and 45 min with secondary antibodies, followed by click chemistry reactions as described below. Slides were mounted in ProLong Gold Antifade Mountant (Molecular Probes). Images were acquired with Zeiss Laser Scanning Confocal Microscope using argon lasers at 488 nm, 543 nm and 633 nm with Zeiss LSM 5 software. Each channel was collected separately, with images at 512 x 512 or 1024 x 1024 pixels, with 4x averaging, without or with a zoom factor. Single confocal sections were acquired or multiple z-sections at 0.2 μM intervals which were then compiled for maximum projection display.

### Homopropargylglycine (HPG) pulse-labeling and click chemistry

From systematic analysis of various parameters we optimised protocols for HPG incorporation, click chemistry and fluorescence detection as follows. Cells on coverslips were mock-infected or infected with HSV-1 by normal procedures (MOI 10). At times indicated, medium was removed and replaced with L-methionine-free DMEM (Sigma-Aldrich) containing 2% FBS for 45 min to deplete methionine prior to the addition of HPG (Molecular Probes) at a final concentration of 0.5 mM for a standard labeling time of 0.5 hr (for microscopy imaging) or 1 mM for 1 hr (for in-gel fluorescence) in L-methionine-free DMEM. In control experiments (e.g. Fig 1C; Fig 2), cells were incubated in either standard media containing methionine (Con) or methionine-free media for 45 min prior to standard media containing methionine (Met). In additional controls, cells were incubated with HPG in the absence or presence of 100 μg/ml CHX, added 1 hr before pulse-labeling with HPG (Fig 1B; Fig 3). For the pre-labeling experiments, methionine depletion and pulse-labeling were performed prior to infection. For pulse-chase analysis, HPG was removed and methionine was reintroduced into the system. When the pulse-labeled cells were to be analysed in parallel for localisation of specific antigens, immunofluorescence with primary and secondary antibodies was carried out as standard (see above). The samples were then subjected to click reaction in a buffer prepared freshly in each case (premixed for 2 min) and containing 10 μM Alexa Fluor 488-azide (Invitrogen); 1 mM CuSO_4_; 10 mM sodium ascorbate; 10 mM amino-guanidine and 1 mM Tris(3-​hydroxypropyltriazolylmethyl)-​amine (TBTA, Sigma-Aldrich) in PBS pH 7.4. The reaction was then allowed to proceed by incubation for 2 hr at RT in the dark. After removal of the reaction cocktail, cells were washed with PBS and mounted on slides in ProLong Gold Antifade Mountant. Images were acquired as described above.

### Quantitative analysis of NPD formation

For quantitation of the relative abundance of HPG-containing newly synthesised protein load within NPDs as a fraction of the signal within the nucleus we used the thresholding and object quantification modules of Image Pro Plus software (Media Cybernetics). HPG-labeled cells were co-stained with DAPI to allow outlining of the entire nucleus and then the background subtracted signal in the HPG-green channel quantified. NPDs were manually outlined and tagged as objects and the signal quantitated for each object and for the total cumulative signal in all objects combined. This was then expressed as a percentage of the entire nuclear signal. The numbers of cells containing induced NPDs was expressed as a percentage of the total population as time progressed, evaluating approximately 100 cells of each time point in each of 3 different experiments. Mean and SD are shown. To enumerate the numbers of NPDs in individual nuclei, a total of 50 nuclei per time point were analysed. The size of each NPDs was measured to the nearest 0.1 μm using Zeiss LSM 5 Image Overlay and Line Measure functions. A total of 50 NPDs per time point were analysed. To assess association of NPDs and PML domains, individual cells were analysed at 4 hr post infection, enumerating maximum projections of each cell for the total number of NPDs, those NPDs immediately juxtaposed to PML (NPD^P^), and total number of PML domains. The raw data shown in S1D Fig is represented in a bar graph showing the average number per cell of total NPD, NPD^P^, and total PML. Similar analyses were performed for NPDs and Hsc70.

### Cytosolic and nuclear fractionation

Cells were washed with ice-cold PBS and removed from 6-well culture dishes using cell scrapers, and centrifuged for 5 min at 1000 rpm. Cell pellets were resuspended and lysed by incubation with 250 μl of PBS extraction buffer (1 mM dithiothreitol (DTT), 1x complete protease inhibitor-EDTA free, 0.5 mM phenylmethanesulfonyl fluoride (PMSF), 0.1 mM sodium orthovanadate, 0.5 mM NaF, and 0.5% Nonidet P-40) for 10 min on ice. 50 μl of sample was retained as total fraction. Cell suspensions were centrifuged at 3000 rpm for 5 min at 4°C. The supernatant representing the cytosolic fraction (200 μl) was retained and nuclear pellet was washed and resuspended in PBS extraction buffer, centrifuged at 3000 rpm for 5 min at 4°C. The washed nuclear pellet was resuspended in 50 μl of PBS extraction buffer (nuclear fraction). All three fractions were made up to a final concentration of 1% SDS in PBS and sonicated prior to click reactions. The nuclear fraction was loaded at 4-fold more of cell equivalents as compared to the total and cytosolic fraction and consequently equal concentrations of proteins (20 μg) were loaded for each track.

### Click chemistry and in-gel fluorescence of newly synthesised proteins

Cells were lysed in PBS containing 2% SDS and diluted to 1% SDS before the click reaction. 100 μg of protein samples were subjected to the click reaction as follows. Click reaction buffers were prepared by adding reagents in the following order with vortex-mixing between the addition of each reagent: capture reagent (IRDye 800CW Azide Infrared Dye from LI-COR, 1 μl, (stock solution 10 mM in DMSO, final concentration 0.1 mM), CuSO_4_ (2 μl, stock solution 50 mM in water, final concentration 1 mM), Tris-(2-Carboxyethyl)phosphine (TCEP, 2 μl, stock solution 50 mM in water, final concentration 1 mM), TBTA (1 μl, stock solution 10 mM in DMSO, final concentration 0.1 mM). Following the addition of the click mixture (6 μl/sample), the samples were placed on a rotating mixer for 1.5 hr at RT, and the reaction was stopped by addition of EDTA to a final concentration of 10 mM. Subsequently, proteins were precipitated (chloroform/methanol, 0.25:1, relative to the sample volume). The precipitated proteins were pelleted by centrifugation at 14,000 rpm for 5 min, washed with methanol and air dried for 10 min. The pellets were then resuspended in 1XSDS sample buffer, boiled for 10 min and 20 μg of proteins were loaded on 12% SDS-PAGE gels. Following electrophoresis, gels were washed with water, fixed in solution containing 40% methanol, 10% acetic acid, 50% water for 5 min and washed with water. In-gel fluorescence detection of translated proteins was performed using a LI-COR Odyssey scanner, and the protein loading was assessed by Coomassie blue staining. Quantitative evaluation of total protein synthesis was assessed by importing scans of the in-gel fluorescence into Quantity One densitometry software (Bio-rad) and using the Plot Profile module for individual lane analysis after background subtraction. The total area under the band peaks was used as a measure of ongoing protein synthesis

### Western blot analysis

Proteins separated by electrophoresis as above were transferred to nitrocellulose membranes which were blocked with Odyssey blocking solution (LI-COR). After blocking, membranes were incubated overnight at 4°C with primary antibodies: mouse anti-ICP4 MAb (1:1500); mouse anti-ICP0 MAb (1:1500); mouse anti-VP5 MAb (Virusys) (1:1500); mouse anti-ICP8 MAb (Abcam) (1:1500) and rabbit anti-ᵞ-tubulin PAb (Sigma) (1:2000); diluted in blocking solution. Membranes were washed three times with 0.05% Tween in PBS and incubated with goat anti-mouse IgG Dylight 680 (Pierce Biotechnology) or goat anti-rabbit IgG Dylight 800 (Pierce Biotechnology) in blocking solution for 1 hr at RT in the dark. Visualisation of protein bands was performed using the LI-COR Odyssey Infrared Imaging System.

## Supporting Information

S1 Fig Quantification of NPD number, size and association with PML domains and Hsc70. (A) The number of cells with HSV-1 induced NPDs is expressed as a percentage of the total number of the cells in three independent time course experiments. 100 cells were analysed at each time point indicated. The mean percentage and SD are shown. (B) The numbers of NPDs per positive nucleus was counted. Each dot represents an individual nucleus on the scatter plot. A total of 50 nuclei per time point indicated were analysed. The mean number of NPDs per nucleus is represented by the red line. (C) The sizes of individual NPDs were measured to the nearest 0.1 μm using Zeiss LSM 5 Image Browser Overlay Function. Approximately 50 individual NPDs were measured at each time point. The mean diameter of the NPDs at each time point is represented by the red line. (D) To assess association of NPDs and PML domains, a total of 10 individual cells (represented by A-J) were analysed at 4 hr post infection. For each cell, the total absolute number of NPDs (green dot), NPDs immediately juxtaposed to PML domains (NPD^P^; orange dot), and total number of PML domains (red dot) were counted. (E) The raw data shown in S1d Fig is represented in a bar graph showing the average number per cell of total NPD, NPD^P^, and total PML. (F) To assess colocalisation of NPDs and Hsc70, a total of 10 cells of each time point indicated were analysed. For each cell, the total number of NPDs (green), NPDs colocalised with Hsc70 foci (NPD^H^; yellow), and total number of Hsc70 foci (red) were counted. Mean and SD are shown.
(TIFF)

S2 Fig NPDs are induced in different cell types by HSV-1 infection. Different cell types as indicated were pulse-labeled for 30 min at 4 hr after mock-infection or HSV-1 infection (MOI 10), fixed and subjected to click chemistry. Diagonal arrows indicate nuclear NPDs formed in different cell types.
(TIF)

S3 Fig Inhibition of proteasome activity does not induce NPD formation in uninfected cells but reveals subtypes of NPDs in infected cells with distinct PML association. Vero cells were pulse-labeled for 30 min at 4 hr after mock-infection (A) or infection (B MOI 10). MG132 (10 μM) was added after the first hour of viral adsorption and was present throughout infection and pulse-labeling. Cells were then fixed and stained for PML, followed by click reaction. The subnuclear localisation of newly synthesised proteins including NPDs (green) and PML (red) were visualised. Vertical arrows in the bottom panels (HSV infected; +MG132) denote a class of PML domains which did not associate with NPDs, while the diagonal arrows (numbered 2) denote a second class of PML domains which colocalised with NPDs. Representative PML class types are labeled on the HPG protein channel. The insert shows an area containing both a class 1 and class 2 domains showing the distinct difference in protein accumulation.
(TIF)

S4 Fig Transcription but not DNA replication is required for the formation of NPDs. Vero cells were pulse-labeled with HPG for 30 min at 4 hr p.i. ACG (10 μM) and Act. D (5 μg/ml) were added after the first hour of viral adsorption and were present throughout infection and pulse-labeling. Cells were fixed and stained for ICP4, followed by click reaction. The subnuclear localisation of newly synthesised proteins including NPDs (green) and ICP4 (red) are indicated.
(TIF)

S5 Fig Proteasome inhibition, heat shock and interferon treatment do not induce the formation of NPDs in uninfected cells. (A) Vero cells were treated with MG132 (10 μM) for 4 hr before pulse-labeling and MG132 maintained during HPG labeling (30 min). Cells were then stained for SUMO and FK2 in parallel with detection of newly synthesised proteins. (B) Vero cells were heat treated at 42°C for 15 min before methionine depletion, and heat treatment continued during depletion and pulse-labeling (30 min). Cells were then fixed and stained for HSP70, followed by click reaction. (C) Vero cells were treated with Interferon-αA/D (5000 U/ml) for 6 hr before HPG-pulse-labeling (30 min) and stained for PML.
(TIFF)

S6 Fig Spatial analysis of newly synthesised proteins in the cytoplasm. Uninfected Vero cells were pulse-labeled for 30 min with 0.5 mM HPG, fixed and simultaneously analysed for newly synthesised proteins by click chemistry and distribution of steady-state organelle markers for the ER (calreticulin, CalR) and Golgi (GM130) as indicated. Vertical arrows denote the relative cytoplasmic localisation of newly synthesised proteins, in relation to steady-state calreticulin, in uninfected. The cytoplasmic localisation of newly synthesised protein shows overlap with the ER.
(TIF)
