# Supplementary figures and images for: Spatial and Temporal Resolution of Global Protein Synthesis during HSV Infection Using Bioorthogonal Precursors and Click Chemistry
Source: PLoS Pathog. 2016 Oct 5;12(10):e1005927. doi: 10.1371/journal.ppat.1005927 (PMC5051704; doi:10.1371/journal.ppat.1005927)

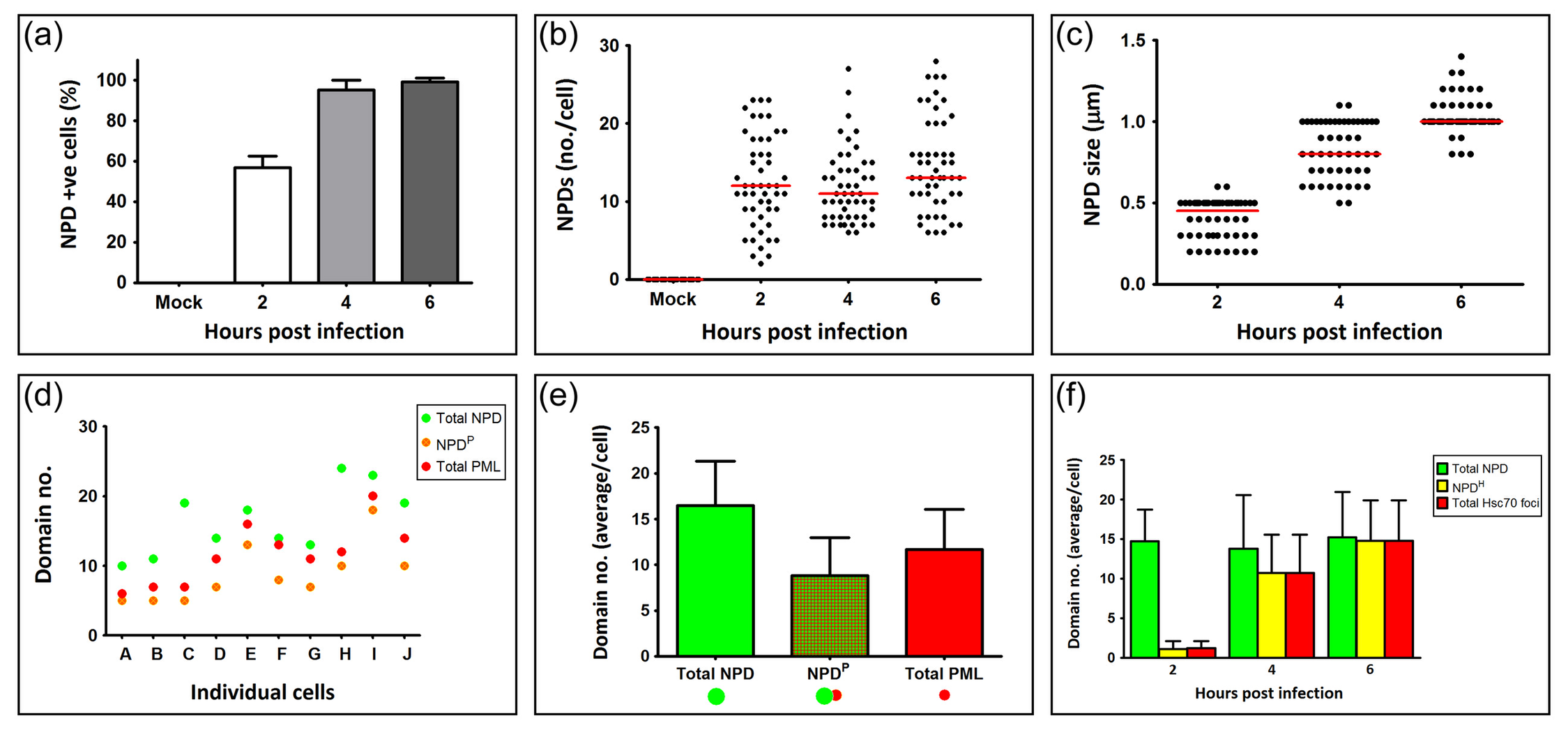

Supplement: S1 Fig — (A) The number of cells with HSV-1 induced NPDs is expressed as a percentage of the total number of the cells in three independent time course experiments. 100 cells were analysed at each time point indicated. The mean percentage and SD are shown. (B) The numbers of NPDs per positive nucleus was counted. Each dot represents an individual nucleus on the scatter plot. A total of 50 nuclei per time point indicated were analysed. The mean number of NPDs per nucleus is represented by the red line. (C) The sizes of individual NPDs were measured to the nearest 0.1 μm using Zeiss LSM 5 Image Browser Overlay Function. Approximately 50 individual NPDs were measured at each time point. The mean diameter of the NPDs at each time point is represented by the red line. (D) To assess association of NPDs and PML domains, a total of 10 individual cells (represented by A-J) were analysed at 4 hr post infection. For each cell, the total absolute number of NPDs (green dot), NPDs immediately juxtaposed to PML domains (NPDP; orange dot), and total number of PML domains (red dot) were counted. (E) The raw data shown in S1d Fig is represented in a bar graph showing the average number per cell of total NPD, NPDP, and total PML. (F) To assess colocalisation of NPDs and Hsc70, a total of 10 cells of each time point indicated were analysed. For each cell, the total number of NPDs (green), NPDs colocalised with Hsc70 foci (NPDH; yellow), and total number of Hsc70 foci (red) were counted. Mean and SD are shown. (TIFF) [file ppat.1005927.s001.tiff]

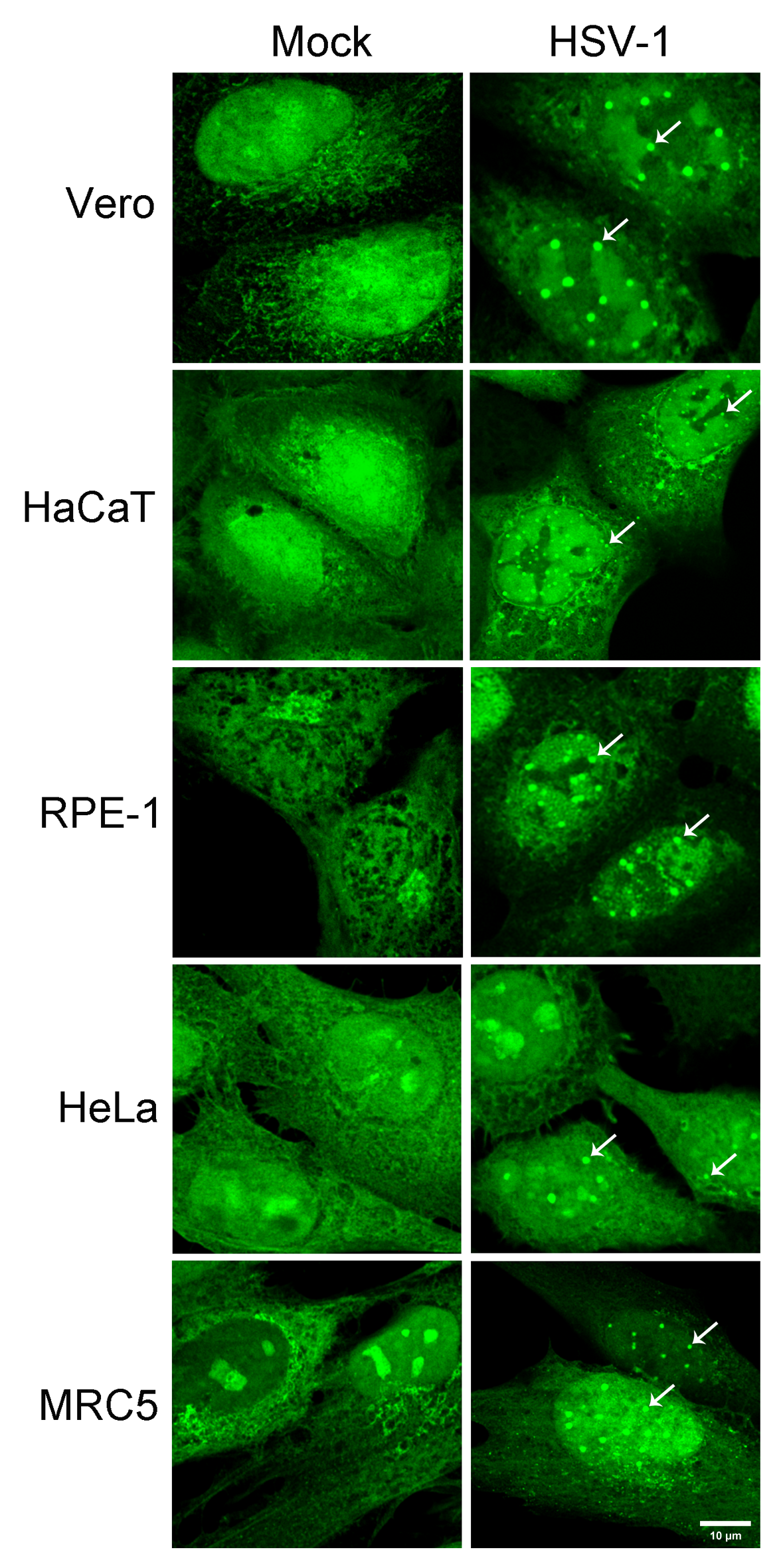

Supplement: S2 Fig — Different cell types as indicated were pulse-labeled for 30 min at 4 hr after mock-infection or HSV-1 infection (MOI 10), fixed and subjected to click chemistry. Diagonal arrows indicate nuclear NPDs formed in different cell types. (TIF) [file ppat.1005927.s002.tif]

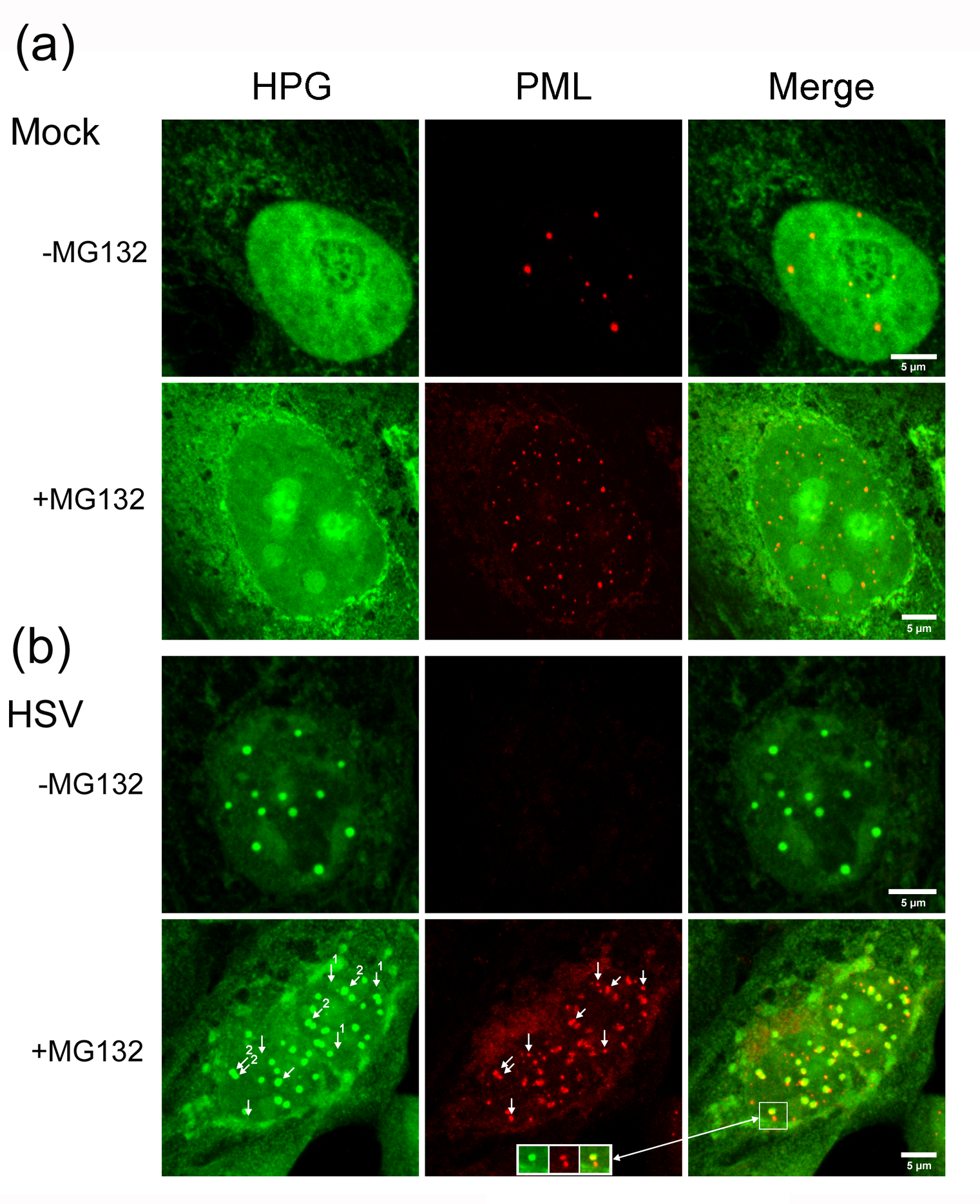

Supplement: S3 Fig — Vero cells were pulse-labeled for 30 min at 4 hr after mock-infection (A) or infection (B MOI 10). MG132 (10 μM) was added after the first hour of viral adsorption and was present throughout infection and pulse-labeling. Cells were then fixed and stained for PML, followed by click reaction. The subnuclear localisation of newly synthesised proteins including NPDs (green) and PML (red) were visualised. Vertical arrows in the bottom panels (HSV infected; +MG132) denote a class of PML domains which did not associate with NPDs, while the diagonal arrows (numbered 2) denote a second class of PML domains which colocalised with NPDs. Representative PML class types are labeled on the HPG protein channel. The insert shows an area containing both a class 1 and class 2 domains showing the distinct difference in protein accumulation. (TIF) [file ppat.1005927.s003.tif]

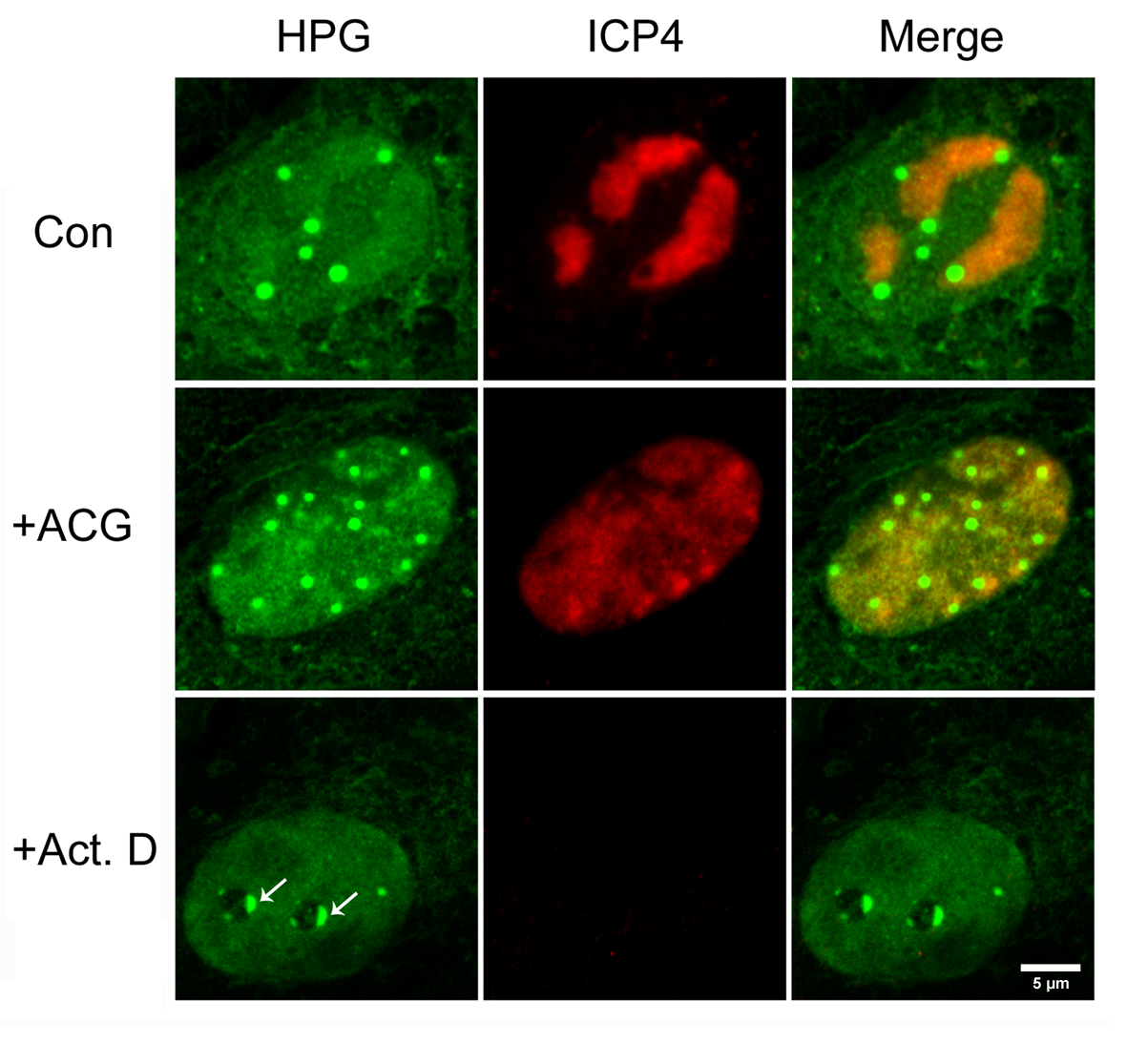

Supplement: S4 Fig — Vero cells were pulse-labeled with HPG for 30 min at 4 hr p.i. ACG (10 μM) and Act. D (5 μg/ml) were added after the first hour of viral adsorption and were present throughout infection and pulse-labeling. Cells were fixed and stained for ICP4, followed by click reaction. The subnuclear localisation of newly synthesised proteins including NPDs (green) and ICP4 (red) are indicated. (TIF) [file ppat.1005927.s004.tif]

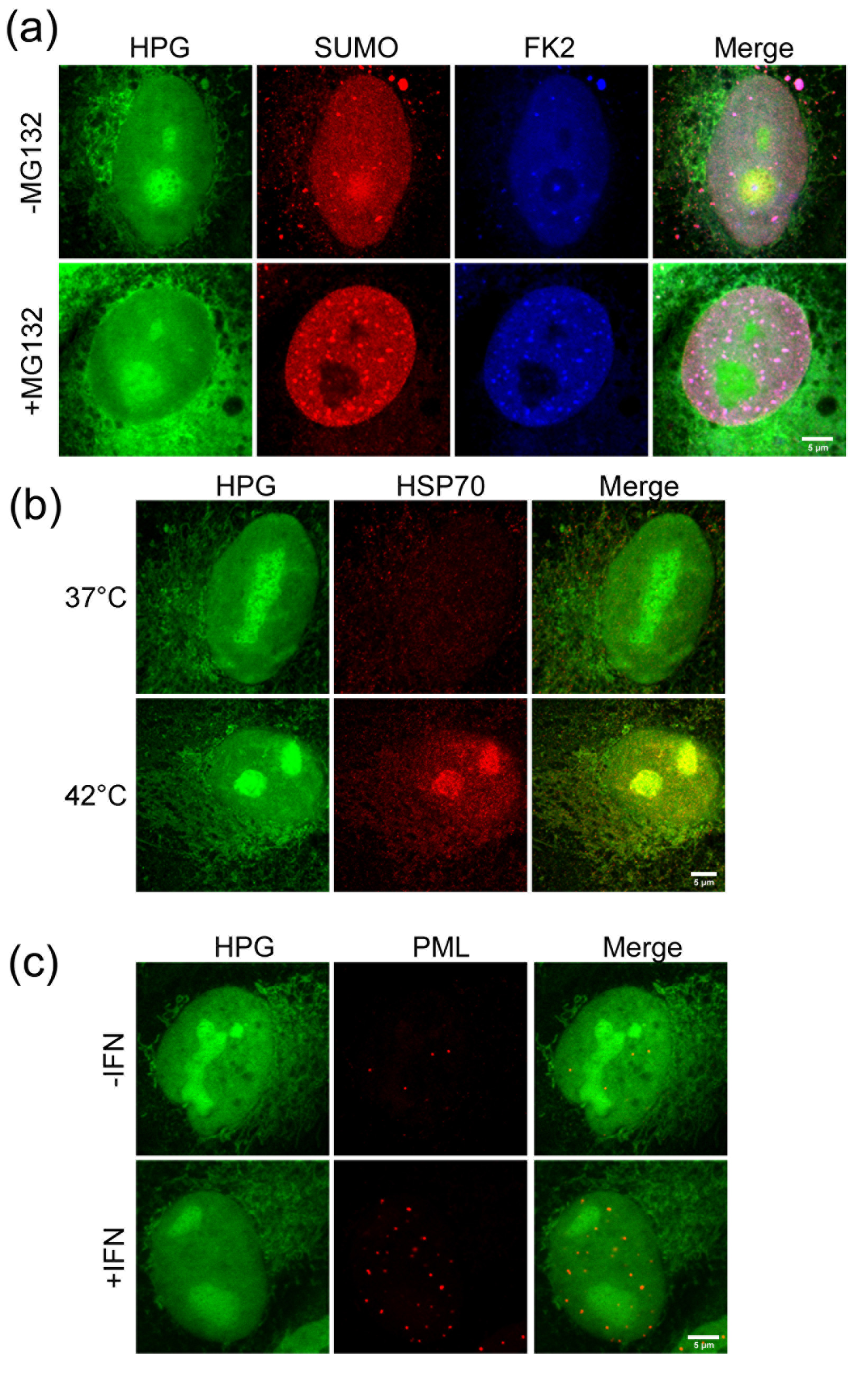

Supplement: S5 Fig — (A) Vero cells were treated with MG132 (10 μM) for 4 hr before pulse-labeling and MG132 maintained during HPG labeling (30 min). Cells were then stained for SUMO and FK2 in parallel with detection of newly synthesised proteins. (B) Vero cells were heat treated at 42°C for 15 min before methionine depletion, and heat treatment continued during depletion and pulse-labeling (30 min). Cells were then fixed and stained for HSP70, followed by click reaction. (C) Vero cells were treated with Interferon-αA/D (5000 U/ml) for 6 hr before HPG-pulse-labeling (30 min) and stained for PML. (TIFF) [file ppat.1005927.s005.tiff]

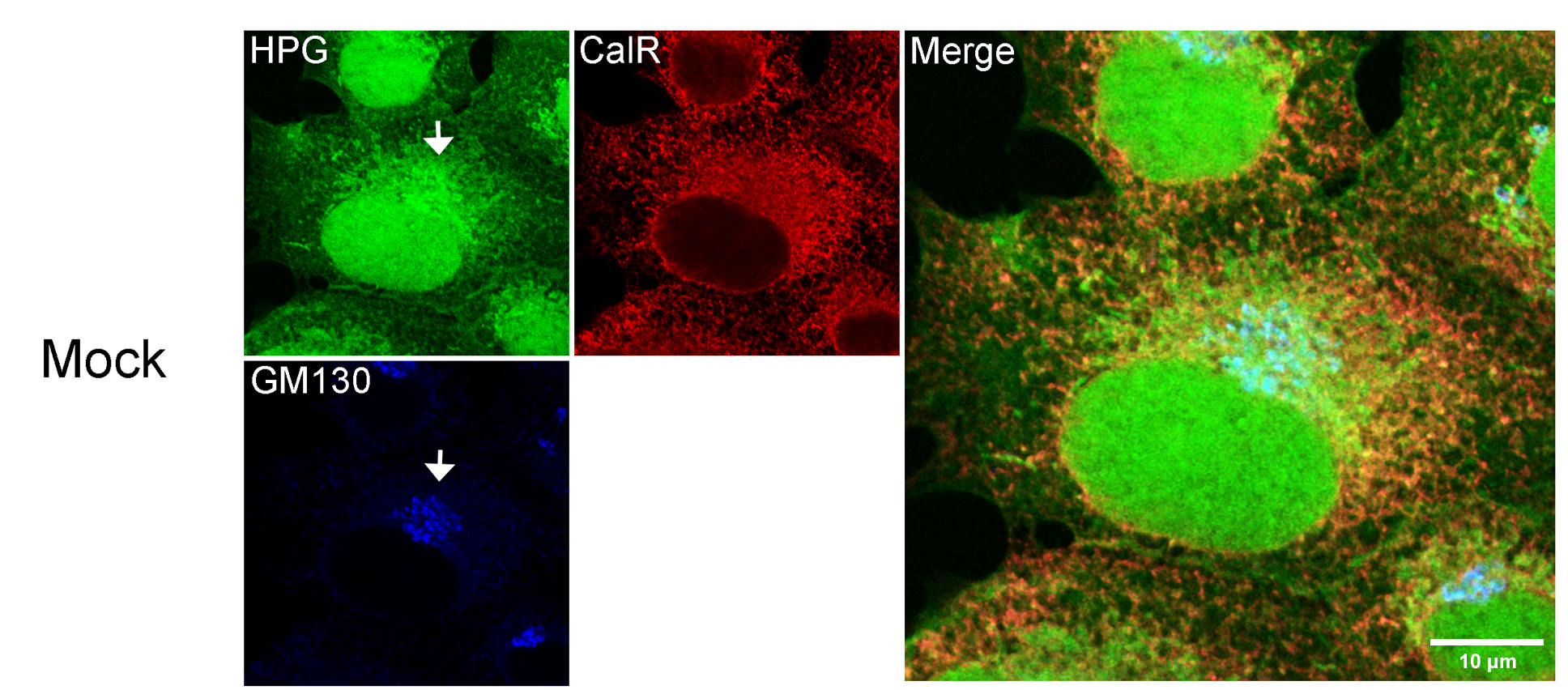

Supplement: S6 Fig — Uninfected Vero cells were pulse-labeled for 30 min with 0.5 mM HPG, fixed and simultaneously analysed for newly synthesised proteins by click chemistry and distribution of steady-state organelle markers for the ER (calreticulin, CalR) and Golgi (GM130) as indicated. Vertical arrows denote the relative cytoplasmic localisation of newly synthesised proteins, in relation to steady-state calreticulin, in uninfected. The cytoplasmic localisation of newly synthesised protein shows overlap with the ER. (TIF) [file ppat.1005927.s006.tif]
